# Exercise Loading Strategies for Patellar Tendinopathy: A Systematic Review and Meta-Analysis of Clinical Outcomes

**DOI:** 10.3390/jfmk11030348

**Published:** 2026-08-31

**Authors:** Alejandro Bruna-Mejias, Rodrigo Cañas-Jamett, Juan José Valenzuela Fuenzalida, María P. Moya-Daza, Gustavo Oyanedel, Gloria Cifuentes-Suazo, Lorena Villaroel, Mathias Orellana Donoso, Eduardo Mateluna-Valls, Juan Jose Cabeza-Salgado, José E. León-Rojas, Juan Sanchis-Gimeno

**Affiliations:** 1 Departamento de Ciencias y Geografía, Facultad de Ciencias Naturales y Exactas, Universidad de Playa Ancha, Valparaíso 2360072, Chile; alejandro.bruna@upla.cl; 2 Departamento de Morfología, Facultad de Medicina, Universidad Andrés Bello, Viña del Mar 2520000, Chile; juan.kine.2015@gmail.com; 3 Physiology Laboratory, Department of Biological Sciences, Faculty of Life Sciences, Universidad Andres Bello, Viña del Mar 2520000, Chile; rcanas@unab.cl; 4 Departamento de Ciencias Química y Biológicas, Facultad de Ciencias de la Salud, Universidad Bernardo O’Higgins, Santiago 8370993, Chile; 5 Facultad de Ciencias de la Salud, Universidad Autónoma de Chile, Santiago 8910060, Chile; maria.moya@uautonoma.cl; 6 Faculty of Health and Social Sciences, University of the Americas, Santiago 8370040, Chile; g.oyanedelamaro@gmail.com; 7 Facultad de Medicina, Carrera de Odontología, Universidad Católica de la Santísima Concepción, Av. Alonso de Ribera 2850, Concepción 4090541, Chile; gbcifuentess@gmail.com; 8 Vicerrectoria Académica, Universidad Mayor, Santiago 7500000, Chile; lorena.villarroel@umayor.cl; 9 Escuela de Medicina, Universidad Finis Terrae, Santiago 7501015, Chile; mathor94@gmail.com; 10 Carrera de Kinesiología, Departamento de Salud, Comunidad y Gestión, Facultad de Ciencias de la Salud, Universidad de Playa Ancha, Valparaíso 2340000, Chile; eduardo.mateluna@upla.cl; 11 Facultad de Medicina, Universidad Católica del Maule, Talca 3460000, Chile; jcabezas@ucm.cl; 12 Cerebro, Emoción y Conducta (CEC) Research Group, Escuela de Medicina, Universidad de las Américas (UDLA), Quito 170521, Ecuador; 13 GIAVAL Research Group, Department of Anatomy and Human Embryology, Faculty of Medicine, University of Valencia, 46001 Valencia, Spain; juan.sanchis@uv.es

**Keywords:** patellar tendinopathy, exercise therapy, tendon loading, eccentric exercise, heavy slow resistance, isometric exercise, resistance training, VISA-P, systematic review, meta-analysis

## Abstract

**Background**: Patellar tendinopathy is a persistent load-related condition in athletes, and exercise is recommended as first-line care; however, the comparative value of specific tendon-loading prescriptions remains uncertain. This systematic review aimed to synthesize exercise-based interventions for adults with patellar tendinopathy, map reported loading prescription parameters, and estimate comparative effects when sufficiently comparable active-loading data were available. **Methods**: We conducted a PROSPERO-registered systematic review following PRISMA 2020. MEDLINE/PubMed, Web of Science Core Collection, Scopus, SPORTDiscus, and CENTRAL via Ovid/EBMR Reviews were searched. Two reviewers independently screened studies, extracted data, and assessed risk of bias. The primary outcome was VISA-P/VISA final score. Randomized active exercise/loading comparisons with extractable final-score data were pooled as mean differences on the original 0–100 scale using random-effects restricted maximum likelihood models with Hartung–Knapp adjustment. **Results**: The searches identified 942 records; 96 full-text reports were assessed and 40 reports were retained for qualitative synthesis. Six active exercise/loading comparisons contributed to the primary comparative synthesis. The pooled mean difference was 5.16 VISA-P/VISA points (95% CI 2.39 to 7.93) in favour of the first-listed intervention, with negligible statistical heterogeneity (tau2 = 0.000; I2 = 0.0%; Q = 3.50, *p* = 0.624). All contributing studies had some concerns in RoB 2, and GRADE certainty was low. **Conclusions**: Structured tendon-loading exercise remains central to rehabilitation. The pooled estimate should be interpreted as a small average comparative signal across clinically distinct active-loading contrasts, not as evidence that one specific loading strategy is universally superior.

## 1. Introduction

Patellar tendinopathy is a chronic insertional overuse condition characterized by pain at the inferior pole of the patella, load-related functional limitation, and structural tendon changes. It is strongly associated with sports that impose repetitive high-magnitude demands on the knee extensor mechanism, particularly jumping, landing, and rapid deceleration. Among elite volleyball and basketball players, point prevalence has been reported at 45% and 32%, respectively, with mean symptom duration exceeding 30 months in affected athletes [1]. Pooled epidemiological data from 28 studies and more than 28,000 participants confirm an overall athletic prevalence of 18%, with the highest burden in volleyball (25%) and basketball (21%) [2]. These data indicate that patellar tendinopathy is not a transient pain complaint but a persistent condition with substantial functional and sporting consequences.

Exercise therapy is widely accepted as a core intervention for patellar tendinopathy, but the optimal way to prescribe and progress tendon loading remains uncertain. Eccentric loading, isometric exercise, heavy slow resistance, and progressive tendon-loading protocols have each been evaluated in clinical trials and systematic reviews [3,4,5]. Previous syntheses support exercise as first-line care and generally find limited added value for several adjunctive treatments when exercise is already provided [4,5]. However, those reviews mainly address whether exercise should be used; whereas, clinicians must also decide how exercise should be dosed, progressed, monitored, and adapted to symptom irritability, sporting phase, and available resources.

Exercise prescription for patellar tendinopathy is a multidimensional loading problem. Relevant parameters include external load, contraction type, volume, frequency, programme duration, tempo, restitution between sessions, pain-monitoring rules, supervision, adherence, and return-to-sport progression. These parameters are not interchangeable, and different trials have tested different clinical questions. For example, progressive tendon-loading exercise produced a greater 24-week VISA-P improvement than eccentric exercise therapy in one randomized trial [6]; whereas, heavy slow resistance at 90% of one-repetition maximum and moderate slow resistance at 55% produced similar clinical, structural, and functional outcomes in another trial [7]. Such findings suggest that improvement may depend on the interaction between loading progression, tolerance, recovery, and context rather than on a single exercise label.

A focused patellar-tendinopathy synthesis is therefore needed to distinguish direct active-loading comparisons from adjunctive, mechanistic, historical, and non-randomized evidence. A broader tendinopathy review with meta-regression found that higher external loads and lower-frequency programmes, generally performed less than daily, were associated with larger effects than daily or more-than-daily schedules, while exercise volume showed less consistent associations [8]. However, only 24 patellar tendinopathy treatment arms contributed to that analysis, and poor dose reporting limited anatomical-site-specific inference [8]. The present review addresses this gap by mapping loading prescription parameters while avoiding inappropriate pooling across evidence streams that answer different clinical questions.

The objective of this systematic review and meta-analysis was to synthesize exercise-based interventions for adults with patellar tendinopathy, map reported loading-strategy and dose-related prescription parameters, and estimate comparative effects for active loading strategies when randomized trials provided sufficiently comparable VISA-P/VISA final-score data. Secondary outcomes included pain, return to sport, satisfaction, adherence, adverse events, and selected functional or performance outcomes.

## 2. Materials and Methods

### 2.1. Study Design and Reporting Framework

This PROSPERO-registered intervention systematic review with meta-analysis was designed to answer two linked questions. First, it synthesized clinical evidence on exercise-based interventions for adults with patellar tendinopathy. Second, it mapped how tendon-loading prescriptions were operationalized across studies, including contraction type, external load, frequency, programme duration, progression, restitution, supervision, adherence, and co-interventions. Quantitative pooling was restricted to randomized active-loading comparisons with compatible VISA-P/VISA final-score data; other evidence streams were retained for clearly labelled qualitative or contextual interpretation.

The protocol was prospectively registered in the International Prospective Register of Systematic Reviews (PROSPERO; CRD420261364763), and the review was conducted and reported in accordance with the Preferred Reporting Items for Systematic Reviews and Meta-Analyses (PRISMA 2020) statement. The PROSPERO record was amended on 19 May 2026 to clarify the focus on exercise-loading prescription parameters and to align the wording of the objective with the finalized synthesis structure. This amendment did not alter the population, intervention domain, primary outcome, eligibility criteria, or the planned use of randomized evidence for the primary quantitative synthesis.

The primary comparative synthesis was planned around randomized controlled trials reporting comparable clinical outcomes. Comparative non-randomized clinical studies, secondary mechanistic reports, and historically relevant exercise studies were considered for qualitative synthesis when they provided relevant information on exercise loading, rehabilitation structure, adherence, safety, or clinical interpretation. Publications derived from the same parent trial were retained as companion papers only when they added contextual, mechanistic, prognostic, or economic information and were not counted as independent clinical effectiveness studies.

### 2.2. Eligibility Criteria

Population: We included adults aged 18 years or older with a clinical diagnosis of patellar tendinopathy. Diagnostic criteria could include activity-related pain localized at or below the inferior pole of the patella, pain provoked by tendon-loading activities such as squatting or jumping, and tenderness on palpation of the inferior patellar pole. Ultrasound or magnetic resonance imaging confirmation was accepted when reported, but was not mandatory. Predominantly chronic cases were eligible, with no minimum symptom duration required. All activity levels were eligible, including elite athletes, recreational athletes, and physically active adults from the general population.

Intervention: Eligible interventions were therapeutic exercise programmes involving loading of the patellar tendon. These included eccentric training, heavy slow resistance training, isometric training, isotonic training, progressive resistance training, low-load blood flow restriction training, whole-body vibration combined with exercise, and multimodal exercise programmes that included patellar tendon loading. Co-administered patient education, activity or load modification, and passive modalities were permitted when applied equally across study arms. Exercise dose variables of interest included intensity, volume, frequency, contraction type, programme duration, progression, tempo, rest, supervision, adherence, and permitted modifications.

Comparator: Eligible comparators included no-exercise control, waitlist, usual care, placebo or sham interventions, alternative exercise programmes with different loading parameters, and other active interventions when the study design allowed the role of exercise loading to be clinically interpreted. For the primary quantitative synthesis, comparisons between exercise programmes with different loading parameters were prioritized over exercise-versus-adjunctive-treatment contrasts. Studies in which both groups received an exercise base but differed by an adjunctive intervention were considered for secondary analyses or narrative synthesis.

Outcomes: The primary outcome was VISA-P. Secondary outcomes included pain intensity, return to sport, satisfaction, adverse events, adherence, and selected functional or performance outcomes, including KOOS, Kujala score, maximal voluntary isometric contraction, countermovement jump, and 5-repetition maximum, when reported.

Study design and publication characteristics: Published randomized controlled trials were prioritized for the primary meta-analysis. Pilot or feasibility randomized trials were eligible if they reported quantitative outcome data. Comparative non-randomized clinical studies and historically relevant exercise studies were retained for qualitative synthesis or sensitivity analyses when they provided clinically relevant information on exercise loading strategies but were not combined with randomized trials in the primary analysis. Only published full-text reports were considered eligible for assessment. Studies published in English, Spanish, Portuguese, French, German, Italian, or Dutch were eligible for inclusion.

Studies were excluded if participants were younger than 18 years, had other concomitant knee pathologies that could confound symptom attribution, previous knee or patellar tendon surgery, injections within 3 months before enrolment, or systemic conditions that could affect tendon health. We also excluded studies in which the intervention did not involve patellar tendon loading, studies in which surgery or injections were the primary intervention without an interpretable exercise comparator, and studies in which invasive co-interventions were administered during the intervention period unless the role of exercise could be interpreted. Additional exclusions were quasi-randomized trials, crossover trials without longitudinal treatment outcomes, observational studies without therapeutic intervention, non-comparative single-arm studies for quantitative synthesis, study protocols without results, conference abstracts without full-text data, animal studies, in vitro studies, and biomechanical studies without clinical outcomes.

### 2.3. Information Sources and Search Strategy

The review was designed to identify published clinical studies evaluating exercise therapy in adults with patellar tendinopathy. The electronic information sources searched were MEDLINE via PubMed, Web of Science Core Collection, Scopus, SPORTDiscus, and CENTRAL via Ovid/EBMR Reviews (Cochrane Central Register of Controlled Trials). MEDLINE/PubMed, Web of Science Core Collection, Scopus, and SPORTDiscus were searched on 9 April 2026, and CENTRAL via Ovid/EBMR Reviews was searched on 01 May 2026. No restriction on publication date or language was applied during electronic searching. Embase was considered during protocol development but was not included in the final search workflow because reproducible access/export could not be implemented within the available review infrastructure. CENTRAL/Ovid was incorporated as an electronic source in the final search workflow; its record handling and contribution to screening are reported in the Results and Supplementary Table S1.

The search strategy combined controlled vocabulary, where available, and free-text terms related to patellar tendinopathy and exercise therapy. The condition component included terms such as patellar tendinopathy, patellar tendinosis, patellar tendonitis, patellar tendinitis, and jumper’s knee. The intervention component included terms related to exercise therapy and tendon-loading exercise, including resistance training, eccentric exercise, isometric exercise, isotonic exercise, heavy slow resistance, progressive tendon loading, and blood flow restriction training. A randomized-trial filter was incorporated to increase specificity for eligible randomized trials, while the full-text screening stage retained clinically relevant comparative exercise studies when they met the broader qualitative synthesis criteria. Search strategies were adapted for each database according to the indexing system and search syntax of the individual platform.

The full search strategies for all searched databases, including CENTRAL/Ovid, are provided in Supplementary Table S1. Reference lists of included studies were screened manually, but no additional eligible reports were identified beyond the electronic database searches.

### 2.4. Selection Process

All records retrieved from the electronic searches were imported into Zotero (version 9.0.6) and deduplicated before screening. Two reviewers independently screened titles and abstracts against the predefined eligibility criteria. Records considered potentially eligible by either reviewer were retrieved for full-text assessment. The same reviewers then assessed the full texts independently to determine final eligibility.

**Figure 1:**
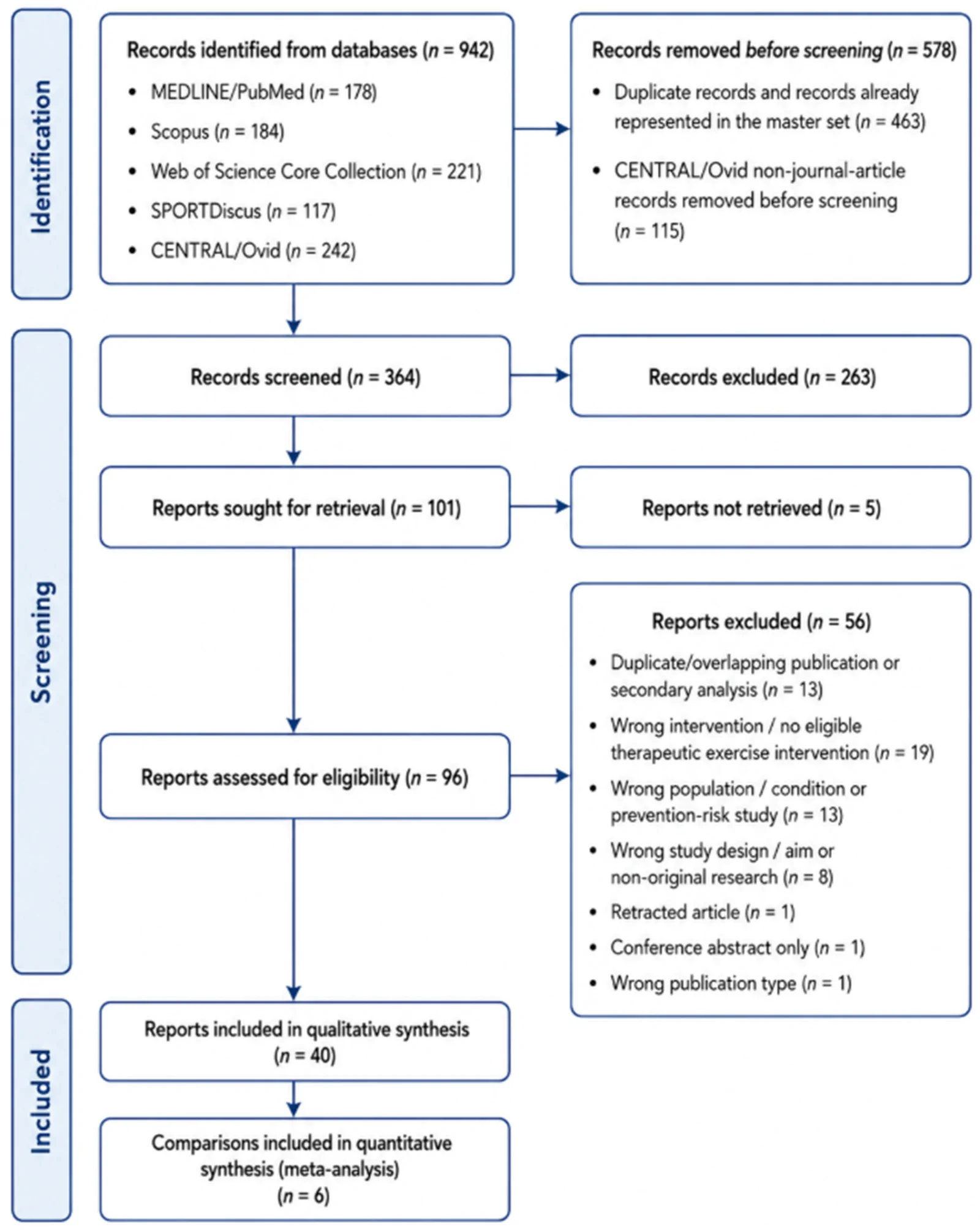
PRISMA 2020 flow diagram for study identification, screening, eligibility assessment, and inclusion. CENTRAL/Ovid records outside the completed journal-article subset were not advanced to title/abstract screening because they were not direct inputs for the completed published clinical-study screening set. The numerical sequence shown in the figure matches the manuscript and Supplementary PRISMA File S1.

Reasons for exclusion were recorded at the full-text stage using standardized PRISMA-compatible categories, including wrong population, wrong intervention, wrong comparator, wrong outcomes, wrong study design, duplicate or overlapping publication, conference abstract only, retracted article, wrong publication type, and full text not retrievable. Any disagreements during title/abstract screening or full-text assessment were resolved by discussion, and a third reviewer was consulted when consensus could not be reached. The study selection process is summarized in the PRISMA 2020 flow diagram (Figure 1).

### 2.5. Data Collection Process

Data were extracted independently by two reviewers using a standardized data extraction form developed a priori for this review. Extracted information included study identification details, country, study design, sample characteristics, participant characteristics, eligibility criteria, intervention characteristics, comparator characteristics, outcome data, adverse events, withdrawals, and funding or conflict-of-interest declarations when reported.

Particular emphasis was placed on exercise prescription variables relevant to the loading-strategy objective of the review, including intensity, volume, frequency, contraction type, programme duration, progression, tempo, rest between sets and sessions, supervision, adherence, co-interventions, and permitted modifications to the exercise programme. Outcome data were extracted for all relevant time points from tables, text, and Supplementary Materials when available.

When required information was unclear or unavailable in the published reports, study investigators were contacted to request additional data. Any discrepancies between reviewers during the extraction process were resolved by discussion, and a third reviewer was consulted when consensus could not be reached.

### 2.6. Risk of Bias Assessment

Risk of bias was assessed independently by two reviewers using the Cochrane Risk of Bias 2 (RoB 2) tool for randomized controlled trials. The assessment considered the following domains: bias arising from the randomization process, bias due to deviations from intended interventions, bias due to missing outcome data, bias in measurement of the outcome, and bias in selection of the reported result. Each domain was judged as low risk of bias, some concerns, or high risk of bias, leading to an overall risk-of-bias judgement for each study outcome.

The primary risk-of-bias assessment was aligned with the main outcome of the review and focused on VISA-P at the primary post-intervention time point whenever possible. Comparative non-randomized or historically relevant studies retained for qualitative synthesis were not combined with randomized trials in the primary risk-of-bias summary for the quantitative synthesis. When required information was unclear or insufficiently reported, study investigators were contacted for clarification. Disagreements between reviewers were resolved by discussion, and a third reviewer was consulted when consensus could not be reached.

For studies retained for narrative, sensitivity, secondary, adjunctive, historical, or mechanistic interpretation but not included in the primary VISA-P/VISA final-score meta-analysis, risk-of-bias assessment was performed according to the intended role of each study in the synthesis. Randomized longitudinal studies considered for sensitivity or secondary analyses were appraised using RoB 2 principles. Non-randomized, quasi-randomized, historical, or comparative clinical studies were classified using a ROBINS-I-oriented methodological appraisal. Acute crossover, mechanistic, imaging, or explanatory studies were not treated as longitudinal clinical effectiveness trials and were therefore not incorporated into the main RoB 2 summary or GRADE rating for the primary outcome.

### 2.7. Outcomes

The primary outcome of the review was VISA-P/VISA final score. VISA-P refers to the Victorian Institute of Sport Assessment-Patella questionnaire, a condition-specific questionnaire scored from 0 to 100, with higher scores indicating better status. The broader VISA-P/VISA wording was retained because some older patellar tendinopathy reports used VISA or related nomenclature for the same 0–100 clinical construct. Outcome data were sought at the end of the intervention and, when available, at longer-term follow-up.

Secondary outcomes were pain intensity, return to sport, satisfaction, adverse events, adherence, and selected functional or performance outcomes. Pain outcomes included visual analogue scale or numerical rating scale measures, including pain assessed during functional tasks when reported. Return-to-sport outcomes included return to pre-injury sport level or time to return to sport. Satisfaction outcomes included global rating or satisfaction categories when reported. Adverse events included exercise-related adverse events and withdrawals due to adverse events. Adherence was defined according to the information reported in the original studies. Functional or performance outcomes included KOOS, Kujala score, maximal voluntary isometric contraction, countermovement jump, and 5-repetition maximum, when available.

### 2.8. Data Synthesis

All included studies were first synthesized qualitatively in structured tables and narrative form. Quantitative meta-analysis was planned when at least two studies reported the same outcome at comparable time points and were considered sufficiently similar in terms of population, intervention, comparator, outcome definition, and follow-up timing.

For continuous outcomes, mean difference was used when studies reported the same scale and standardized mean difference was used when different instruments measured the same construct. For dichotomous outcomes, risk ratios or odds ratios with 95% confidence intervals were planned. Random-effects meta-analysis was prespecified for all pooled analyses.

Statistical heterogeneity was assessed using the I2 statistic, tau2, and Cochran’s Q test. Prespecified subgroup analyses were planned according to contraction type, programme duration, exercise frequency, exercise intensity, participant activity level, comparator type, and risk of bias. Meta-regression was considered for dose–response questions only if the number of comparisons was adequate. For the primary outcome, only six comparisons were available; therefore, subgroup analyses and meta-regression were not conducted because they would have been statistically unstable and clinically over-interpretable. Formal sensitivity analyses were also not conducted for the primary outcome because the non-primary studies differed in outcome structure, reporting format, unit of analysis, comparator type, or extractability. These studies were synthesized narratively or reserved for separate exploratory analyses.

For the primary comparative synthesis, VISA-P/VISA final scores were analyzed as between-group mean differences on the original 0–100 scale. Final-score mean differences were prioritized because the principal quantitative question was comparative: whether one active loading strategy produced better endpoint status than another in randomized trials. Within-group changes were not pooled because they are vulnerable to natural history, regression to the mean, contextual effects, baseline severity, and differences in follow-up duration. Effect estimates were calculated as the mean final score of the first-listed intervention minus the mean final score of the second-listed intervention; therefore, positive values favoured the first-listed loading strategy. This direction of effect was used for analytical consistency across heterogeneous active comparisons and should not be interpreted as implying that the first-listed strategy represents a clinically preferred category across all trials. When studies reported standard errors of the mean, standard deviations were derived using SD = SEM x square root of *n*. For multi-arm trials, a single clinically prespecified contrast was selected for the primary synthesis to avoid double-counting participants. The random-effects model was fitted using restricted maximum likelihood with Hartung–Knapp adjustment in R version 2026.04.0+526, using the metafor package version 5.0-1.

### 2.9. Reporting Bias and Certainty of Evidence

Risk of bias due to missing results was planned to be assessed for outcomes included in quantitative synthesis. When at least 10 studies contributed to a meta-analysis, and when study sizes were sufficiently variable to make such methods informative, funnel plots and appropriate tests for funnel plot asymmetry were planned to explore the possibility of small-study effects and missing evidence. These analyses were to be interpreted cautiously and in conjunction with visual inspection, recognizing that funnel plot asymmetry is not specific to publication or reporting bias and may reflect alternative explanations. Where appropriate, contour-enhanced funnel plots and additional sensitivity analyses were considered to aid interpretation. Publication bias was not formally assessed for the current primary meta-analysis because fewer than 10 comparisons contributed to the synthesis; therefore, small-study effects remain an interpretive limitation.

The certainty of the evidence was evaluated using the Grading of Recommendations Assessment, Development and Evaluation (GRADE) approach. For the primary VISA-P/VISA final-score outcome, the evidence body started at high certainty because the synthesis was based on randomized controlled trials. Certainty was rated across the domains of risk of bias, inconsistency, indirectness, imprecision, and publication bias. Secondary outcomes were not assigned pooled GRADE ratings because they were not synthesized quantitatively in a sufficiently comparable manner across studies, mainly due to heterogeneous outcome metrics, follow-up timing, intervention contrasts, and incomplete reporting. The final Summary of Findings assessment presents the number of studies and participants, the pooled effect estimate, the domain-level GRADE judgements, and the overall certainty rating for the primary outcome.

## 3. Results

### 3.1. Study Selection

The electronic database searches identified 942 records, including 700 records from MEDLINE/PubMed, Scopus, Web of Science Core Collection, and SPORTDiscus and 242 records from CENTRAL/Ovid. After removal of 463 duplicates or records already represented in the master set, and after removing 115 CENTRAL/Ovid records outside the completed journal-article subset before screening, 364 records were screened by title and abstract. Of these, 263 records were excluded at title/abstract level. A total of 101 reports were sought for retrieval. Five reports could not be retrieved despite targeted retrieval attempts, leaving 96 full-text reports for eligibility assessment (Figure 1).

Of the 96 full-text reports assessed, 56 were excluded with PRISMA-compatible reasons. The main reasons were duplicate or overlapping publications/secondary analyses (*n* = 13), wrong intervention or no eligible therapeutic exercise intervention (*n* = 19), wrong population, wrong condition, or prevention-risk design (*n* = 13), wrong study design, wrong aim, or non-original research (*n* = 8), retracted article (*n* = 1), conference abstract only (*n* = 1), and wrong publication type (*n* = 1). Forty reports were retained for qualitative synthesis. The 11 unique CENTRAL/Ovid journal-article records identified after deduplication were excluded at title/abstract screening and did not undergo full-text assessment. Article-level full-text exclusion reasons are provided in Supplementary Table S2.

### 3.2. Characteristics of Reports Included in the Qualitative Synthesis and Final Synthesis Role

Forty reports were retained for qualitative synthesis and organized by analytical contribution rather than treated as one homogeneous evidence set. Reports directly comparing loading strategy, loading intensity, contraction type, progression, frequency, or restitution were prioritized for quantitative extraction. Six randomized exercise/loading comparisons provided extractable VISA-P/VISA final-score data and were included in the primary meta-analysis.

The remaining reports informed secondary, sensitivity, or narrative interpretation. These included adjunctive interventions over a common exercise base, acute or mechanistic loading experiments, historical comparative exercise studies, non-randomized or quasi-randomized clinical studies, and reports with incomplete numerical outcome data. When required data were unavailable and no author response was obtained, fields were coded as NR/no author response and were not imputed.

For readability, the retained evidence can be understood in three levels: primary quantitative evidence from randomized active loading comparisons; secondary or non-pooled clinical evidence addressing adjunctive, surgical, or less directly comparable rehabilitation contrasts; and contextual evidence from acute, mechanistic, historical, or non-randomized studies. This structure was used to preserve clinical interpretability and avoid treating distinct research questions as a single homogeneous evidence base. The characteristics, evidence type, intervention contrast, final synthesis role, and quantitative handling of the retained reports are summarized in Table 1.

#### Exercise Loading and Dose Parameters

Exercise prescription was treated as a multidimensional construct that included loading strategy, contraction type, frequency, volume, intensity, programme duration, progression, tempo, supervision, between-session rest, co-interventions, and adherence. The main loading categories were eccentric decline-squat training, heavy or moderate slow resistance training, progressive tendon-loading exercise, isometric or isotonic loading, low-load blood-flow restricted training, and exercise combined with whole-body vibration.

The finalized dose table reports only information available in published reports or verified during extraction. Missing dose, adherence, or numerical outcome information was not imputed. This conservative approach preserves transparency and prevents overstatement of dose–response inference. The exercise-loading and dose parameters of the longitudinal clinical interventions are summarized in Table 2.

### 3.3. Risk of Bias

Risk of bias was assessed for the six randomized trials contributing to the primary VISA-P/VISA final-score meta-analysis. The assessment was outcome-specific and focused on the follow-up time point used in the primary synthesis. All six studies were judged as having some concerns overall; none was rated as high risk of bias for the primary outcome.

The most recurrent concerns were the inability to blind participants and therapists in exercise-based interventions, the self-reported nature of VISA-P/VISA, incomplete reporting of allocation concealment in some trials, and limited protocol or statistical analysis plan detail in older or smaller studies. These judgments support inclusion of the studies in the primary synthesis while requiring cautious interpretation of the pooled effect. Visual summaries of the outcome-level RoB 2 judgements are provided in Supplementary Figures S1 and S2, whereas detailed role-specific appraisals of studies outside the primary meta-analysis are reported in Supplementary Table S3. Study-level RoB 2 judgements for the six trials contributing to the primary comparative synthesis are presented in Table 3.

### 3.4. Quantitative Synthesis

Six active exercise/loading comparisons were included in the primary comparative final-score synthesis of VISA-P/VISA. The outcome was analyzed as a between-group mean difference on the original 0–100 scale, with positive values favouring the first-listed intervention in each comparison. Using a random-effects REML model with Hartung–Knapp adjustment, the pooled mean difference was 5.16 points (95% CI 2.39 to 7.93), with negligible statistical heterogeneity (tau2 = 0.000; I2 = 0.0%; Q = 3.50, *p* = 0.624).

This estimate should be interpreted as an average across trial-specific active loading contrasts, not as direct evidence that any single exercise-loading strategy is universally superior. The analysis used randomized between-group endpoint differences to preserve comparative inference. However, several trial arms improved substantially from baseline, and those within-group changes are clinically relevant for understanding exercise responsiveness. They were not pooled because they cannot distinguish treatment effects from natural history, regression to the mean, contextual effects, baseline differences, or unequal endpoint timing.

**Figure 2:**
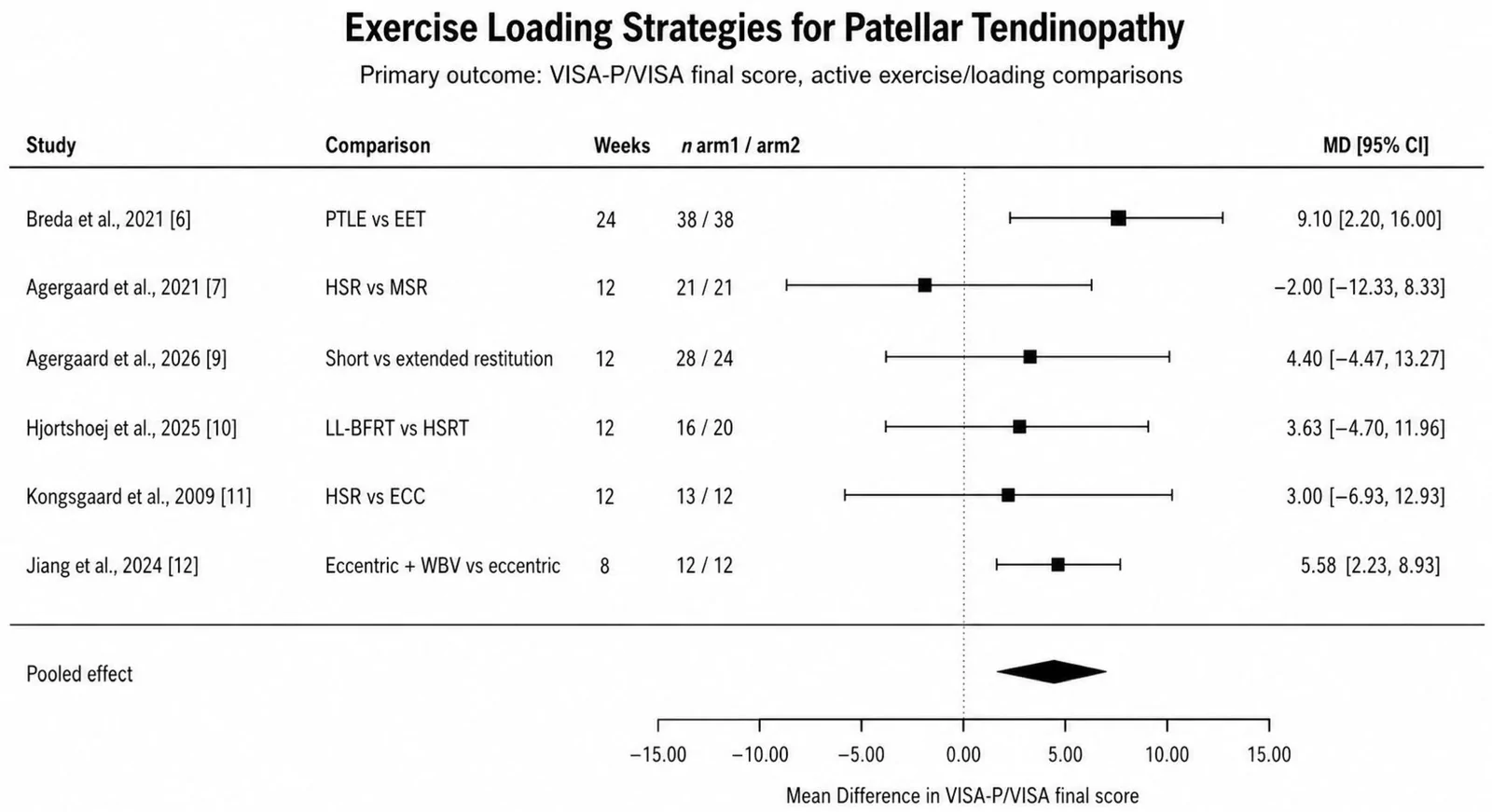
Forest plot of active exercise/loading comparisons for VISA-P/VISA final score in patellar tendinopathy. The studies represented are Breda et al. [6], Agergaard et al. [7,9], Hjortshoej et al. [10], Kongsgaard et al. [11], and Jiang et al. [12]. Effect estimates are mean differences (MDs), calculated as the mean final score of the first-listed intervention minus the mean final score of the second-listed intervention. Positive values favour the first-listed intervention; whereas, negative values favour the second-listed intervention. This direction of effect is an analytical convention for each trial-specific contrast and should not be interpreted as a universal hierarchy of loading strategies. Endpoint timing ranged from 8 to 24 weeks; therefore, the pooled estimate should not be interpreted as a time-standardized treatment effect. The random-effects model was fitted using restricted maximum likelihood with Hartung–Knapp adjustment. The pooled effect was MD = 5.16 points (95% CI 2.39 to 7.93), with tau2 = 0.000, I2 = 0.0%, Q = 3.50, and *p* = 0.624. PTLE, progressive tendon-loading exercise; EET, eccentric exercise therapy; HSR, heavy slow resistance; MSR, moderate slow resistance; LL-BFRT, low-load blood-flow restriction training; HSRT, heavy slow resistance training; ECC, eccentric exercise; WBV, whole-body vibration.

Endpoint timing ranged from 8 to 24 weeks (Table 4). Accordingly, the pooled estimate should be read as a conservative comparative summary across active-loading trials rather than as a time-standardized treatment effect. Although statistical heterogeneity was negligible, clinical heterogeneity across comparisons remains important because the synthesis combines distinct questions involving progression strategy, external load intensity, restitution/frequency, blood-flow restriction, heavy slow resistance, eccentric loading, and vibration added to exercise. The study-level effects and the pooled comparative estimate are presented in Figure 2.

### 3.5. Certainty of Evidence for the Primary Outcome

Using GRADE, the certainty of evidence for the primary VISA-P/VISA final-score outcome was rated as low. The rating was downgraded for risk of bias and indirectness. The certainty of evidence was downgraded for indirectness because the pooled estimate combined clinically distinct active loading contrasts, including differences in progression strategy, external load, frequency/restitution, blood-flow restriction, heavy slow resistance, eccentric loading, and vibration-assisted exercise. No downgrade was applied for inconsistency or imprecision. Publication bias was not formally assessed because fewer than 10 comparisons contributed to the primary meta-analysis; therefore, small-study effects remain an interpretive limitation. The domain-level GRADE assessment and overall certainty rating are summarized in Table 5, and the complete GRADE evidence profile is provided in Supplementary Table S4.

### 3.6. Narrative and Secondary Synthesis

Thirty-four retained reports did not contribute to the primary comparative final-score synthesis. They were used only according to their prespecified evidentiary role, as summarized in Table 1 and detailed in Supplementary Table S3. The most common reasons for non-pooling were adjunctive contrasts over a common exercise base, non-exercise or surgical comparators, acute or mechanistic designs, historical or non-randomized designs, incomplete numerical extraction, figure-derived data, median/IQR or change-score reporting, tendon-level analysis, and incompatible outcome or follow-up structures.

These studies were not merged with the six active loading comparisons because doing so would have changed the review question and inflated the apparent precision of the primary estimate. Adjunctive studies addressed whether interventions such as extracorporeal shockwave therapy, dry needling, laser therapy, therapeutic ultrasound, taping, injection, supplementation, manual therapy, or traditional therapies added benefit to an exercise programme, rather than whether one exercise-loading prescription was superior to another. Acute and mechanistic studies informed biological plausibility and short-term responses, but they did not provide longitudinal therapeutic effectiveness estimates.

Historical and non-randomized studies contextualized the evolution of eccentric decline-board, concentric, isometric, and resistance-loading approaches, but they were not used as primary causal evidence. Across the broader retained evidence base, adherence and adverse-event reporting were heterogeneous; therefore, feasibility and safety were summarized narratively rather than pooled.

Overall, the qualitative evidence supports viewing exercise therapy for patellar tendinopathy as a multidimensional loading intervention. The available data suggest that structured, progressive, and clinically monitored tendon-loading programmes can improve symptoms and function, but they do not justify a single universal loading prescription or a definitive hierarchy among individual dose components.

## 4. Discussion

This systematic review and meta-analysis synthesized exercise-based interventions for adults with patellar tendinopathy and mapped how loading prescriptions were reported across the available clinical literature. The primary comparative synthesis included six randomized active-loading comparisons and showed a small average endpoint advantage favouring the first-listed intervention. The certainty of evidence was low, mainly because all contributing studies had some concerns in the outcome-specific RoB 2 assessment and because the pooled estimate combined clinically distinct active-loading contrasts. Therefore, the main conclusion is not that one loading protocol is superior, but that current evidence supports structured tendon-loading exercise while remaining insufficient to establish a universal hierarchy of loading prescriptions.

The clinical interpretation of the pooled effect requires caution. Several included trials reported meaningful within-group improvements in both exercise arms, which supports the responsiveness of patellar tendinopathy to structured rehabilitation. However, within-group change does not isolate comparative treatment effects because it may reflect natural history, regression to the mean, contextual effects, baseline severity, adherence, and follow-up duration. For this reason, the between-group final-score mean difference was retained as the primary comparative estimator, while being interpreted as a conservative summary across non-identical active-loading contrasts with endpoints ranging from 8 to 24 weeks.

These findings are consistent with previous syntheses showing that exercise is central to patellar tendinopathy management [3,4,5]. Earlier reviews have supported eccentric loading, isometric exercise, heavy slow resistance, and progressive tendon-loading approaches, while also finding limited evidence that adjunctive treatments add clear benefit when exercise is already provided [3,4,5]. The present review refines that evidence by separating direct active-loading comparisons from adjunctive, mechanistic, historical, and non-randomized evidence streams, thereby reducing the risk of treating different clinical questions as a single homogeneous body of evidence.

The randomized trials included in the primary synthesis illustrate why a single prescription cannot currently be endorsed. Progressive tendon-loading exercise may offer advantages over traditional eccentric exercise when progression is staged from isometric to isotonic, energy-storage, and sport-specific loading according to pain-based criteria [6]. In contrast, heavy and moderate slow resistance produced similar improvements when volume was matched [7], extended restitution did not enhance 12-week exercise-based outcomes compared with a shorter-restitution structure [9], and low-load blood-flow restricted training produced broadly comparable outcomes to heavy slow resistance [10]. These results suggest that load magnitude, contraction type, frequency, restitution, and adjunctive stimuli should be interpreted in relation to tolerance, progression, supervision, sport demands, and feasibility rather than as isolated determinants of success.

The broader dose–response literature provides useful but still provisional context. Pavlova et al. reported larger pooled effects for interventions using additional external resistance and for lower-frequency programmes, generally performed less than daily, compared with daily or more-than-daily schedules across several tendinopathy locations [8]. However, the patellar tendinopathy subset was limited, and dose reporting was inconsistent [8]. In the present patellar-specific review, the number of directly comparable trials was too small for subgroup analysis or meta-regression. The dose-related findings should therefore be viewed as hypothesis-generating rather than confirmatory evidence for a precise loading rule.

For practice, the most defensible recommendation is to prescribe tendon-loading exercise as an individualized, progressive, and clinically monitored intervention. Progressive tendon-loading frameworks may be useful when clinicians need staged reintroduction of energy-storage and sport-specific demands [6]. Heavy or moderate slow resistance may be appropriate when controlled and quantifiable loading is feasible [7]. Low-load blood-flow restriction may be considered when high external loads are poorly tolerated, although superiority over heavy slow resistance has not been established [10]. Adjunctive or mechanistic approaches should be used cautiously unless their independent added value over exercise alone is demonstrated [5,12].

This review has several strengths. It was prospectively registered, reported according to PRISMA 2020, used a transparent article-level selection process, incorporated CENTRAL/Ovid into the final search workflow, applied outcome-specific RoB 2 assessment, used GRADE for the primary outcome, avoided double-counting in multi-arm trials, and separated primary comparative evidence from adjunctive, mechanistic, historical, and non-primary evidence. This structure preserves clinical interpretability while allowing the broader literature to inform context without overextending causal claims.

Several limitations should be acknowledged. The primary comparative synthesis included only six active-loading comparisons, preventing reliable subgroup analysis or meta-regression. Although statistical heterogeneity was negligible, the pooled comparisons differed clinically in prescription structure, comparator, and endpoint timing. Embase was not searched because reproducible access/export could not be implemented within the available review infrastructure. The CENTRAL/Ovid search used the completed journal-article subset for deduplication and title/abstract screening, which may have limited retrieval of unpublished or non-journal records. Several clinically relevant studies could not be pooled because of incompatible outcome structures, change-score reporting, median/IQR data, figure-derived data, non-exercise comparators, overlapping publications, or numerical data not available in a directly extractable format. Adherence, safety, and publication bias also remain interpretive limitations because reporting was inconsistent and fewer than 10 comparisons contributed to the primary synthesis.

Future trials should report exercise prescription with sufficient precision to support cumulative synthesis. At minimum, studies should describe intensity, volume, frequency, contraction type, progression rules, pain-monitoring criteria, supervision, adherence, co-interventions, adverse events, return-to-sport criteria, and extractable outcome data at prespecified time points. Trials should also distinguish short-term analgesic responses from sustained clinical recovery and avoid conflating adjunctive treatment effects with the effect of loading strategy itself. Such reporting would allow future reviews to test whether specific dose components, rather than broad exercise labels, explain clinically meaningful differences in recovery.

## 5. Conclusions

In adults with patellar tendinopathy, the primary comparative final-score synthesis showed a small average difference in VISA-P/VISA, but the certainty of evidence was low because of risk-of-bias concerns and indirectness across clinically distinct active-loading contrasts. Structured tendon-loading exercise should remain central to rehabilitation, yet the current evidence does not support a single universally superior loading prescription. Clinical decisions should be individualized according to symptoms, load tolerance, functional goals, sport demands, supervision, and feasibility, while future trials should report dose and progression parameters with enough precision to support stronger comparative synthesis.

### Key Points

Findings: Active exercise/loading comparisons showed a small average endpoint difference in VISA-P/VISA final score, but the effect was modest and supported by low-certainty evidence.

Implications: Exercise prescription should be viewed as an individualized, structured, and progressive loading process rather than as the selection of one universally superior protocol.

Caution: Adjunctive, mechanistic, acute, historical, and clinically distinct loading studies should not be merged with active loading-strategy trials when estimating comparative clinical effectiveness, and negligible statistical heterogeneity should not be interpreted as full clinical homogeneity.

## Figures and Tables

**Table 1:** Characteristics of reports retained for qualitative synthesis and final role in the review.

Study	Design/Evidence Type	Intervention/Comparison	Final Role in Review	Quantitative Handling
Breda et al., 2021 [6]	RCT	Progressive tendon-loading exercise vs. eccentric exercise therapy	Primary quantitative synthesis	Included in main VISA-P/VISA final-score meta-analysis
Agergaard et al., 2021 [7]	RCT	Heavy slow resistance vs. moderate slow resistance	Primary quantitative synthesis	Included in main VISA-P/VISA final-score meta-analysis
Agergaard et al., 2026 [9]	RCT	Short restitution/higher frequency vs. extended restitution/lower frequency	Primary quantitative synthesis	Included in main VISA-P/VISA final-score meta-analysis
Hjortshoej et al., 2025 [10]	RCT	Low-load blood-flow restriction training vs. heavy slow resistance training	Primary quantitative synthesis	Included in main VISA-P/VISA final-score meta-analysis
Kongsgaard et al., 2009 [11]	Randomized single-blind trial	Corticosteroid injection vs. eccentric decline squat vs. HSR	Primary quantitative synthesis for exercise arms	HSR vs. eccentric arms included; injection arm excluded from main exercise-only synthesis
Jiang et al., 2024 [12]	Four-arm RCT	Eccentric training vs. eccentric training plus WBV at 30, 40, or 50 Hz	Primary quantitative synthesis	50 Hz + eccentric vs. eccentric alone included to avoid double counting of the control group
Ruffino et al., 2021 [13]	RCT	Inertial flywheel resistance vs. heavy slow resistance	Sensitivity/narrative candidate	Not included in main analysis; numerical data required figure-derived extraction
Jonsson and Alfredson, 2005 [14]	Prospective randomized trial	Eccentric decline-board training vs. concentric training	Sensitivity/narrative candidate	Not included in main analysis; unit-of-analysis and attrition concerns
Stasinopoulos et al., 2012 [15]	Controlled clinical trial	Eccentric training plus static stretching vs. eccentric training alone	Secondary/sensitivity candidate	Not included in main analysis; non-randomized/alternate allocation concerns and incomplete compatible data
van Ark et al., 2016 [16]	RCT	Isometric exercise vs. isotonic exercise	Narrative/sensitivity candidate	Not included in main analysis; short follow-up and median/IQR reporting
Frohm et al., 2007 [17]	Pilot RCT	Eccentric overload device vs. decline-board eccentric training	Narrative/sensitivity candidate	Not included in main analysis; small pilot and non-compatible summary data
Rieder et al., 2022 [18]	Randomized equivalence trial	Whole-body vibration vs. HSR vs. waitlist control	Narrative or separate change-score analysis	Not included in main final-score analysis; change-score structure and comparator heterogeneity
Bahr et al., 2006 [19]	RCT	Open tenotomy vs. eccentric decline-board training	Separate exercise vs. surgery synthesis	Not pooled with active exercise/loading comparisons
Purdam et al., 2004 [20]	Pilot comparative study	Standard single-leg squat vs. decline-board eccentric squat	Historical/narrative evidence	Not included in VISA-P synthesis because VISA/VISA-P was not reported
Fendri et al., 2026 [21]	RCT	Progressive tendon-loading eccentric exercise vs. usual sport/activity continuation	Narrative only; extractable numerical data unavailable after author contact	Not pooled; verified numerical data unavailable/no author response
Magendran et al., 2025 [22]	Randomized experimental study	Plyometric training vs. aquatic training	Narrative candidate	Not pooled; intervention/outcome comparability limitations and no directly extractable data after author contact
Xiao et al., 2024 [23]	RCT	HSR plus therapeutic ultrasound vs. HSR vs. ultrasound	Secondary/adjunctive synthesis	Not pooled in main analysis; adjunctive contrast over exercise base
Ferreira et al., 2025 [24]	RCT	Dynamic Tape plus progressive tendon-loading exercise vs. sham tape plus exercise	Secondary/adjunctive synthesis	Not pooled in main analysis; adjunctive contrast over common exercise base
Sannasi et al., 2025 [25]	RCT	Fascial manipulation vs. eccentric training plus stretching	Narrative/secondary clinical evidence	Not pooled in main exercise-loading synthesis; comparator not isolated loading strategy
Zhao et al., 2022 [26]	RCT	Baduanjin training plus shockwave vs. shockwave alone	Secondary/adjunctive synthesis	Not pooled in main analysis; exercise combined with adjunctive intervention
Sanchez-Gomez et al., 2022 [27]	Pilot RCT	HMB supplementation plus rehabilitation vs. placebo plus rehabilitation	Secondary/narrative synthesis	Not pooled in main analysis; nutrition/supplement adjunct over rehabilitation
Sharif et al., 2022 [28]	RCT	Ultrasound-guided dry needling plus conventional physical therapy vs. conventional physical therapy	Secondary/adjunctive synthesis	Not pooled in main analysis; adjunctive intervention
Stepanenko et al., 2022 [29]	Randomized clinical study	Exercise rehabilitation plus laser vs. exercise rehabilitation alone	Secondary/adjunctive synthesis	Not pooled in main analysis; laser adjunct contrast
Lopez-Royo et al., 2021 [30]	RCT	Dry needling plus eccentric exercise vs. PNE plus eccentric exercise vs. sham needling plus eccentric exercise	Secondary/adjunctive synthesis	Not pooled in main analysis; needling/PNE adjunct contrast over common eccentric base
Olesen et al., 2021 [31]	Randomized placebo-controlled trial	IGF-1 injection plus HSR vs. saline plus HSR	Secondary/adjunctive synthesis	Not pooled in main analysis; injection adjunct over common HSR
Lee et al., 2020 [32]	RCT	Eccentric decline squat vs. eccentric exercise plus ESWT	Secondary/adjunctive synthesis	Not pooled in main analysis; exercise plus ESWT contrast
Thijs et al., 2017 [33]	Randomized placebo-controlled trial	ESWT plus eccentric training vs. sham ESWT plus eccentric training	Secondary/adjunctive synthesis	Not pooled in main analysis; ESWT/sham contrast over common exercise
Abat et al., 2016 [34]	RCT	USGET plus eccentric exercise vs. conventional electrophysiotherapy plus eccentric exercise	Secondary/adjunctive synthesis	Not pooled in main analysis; adjunctive/interventional contrast
Zhao et al., 2016 [35]	RCT	Moxibustion plus exercise prescription vs. instrumental therapy plus exercise prescription	Secondary/narrative synthesis	Not pooled in main analysis; adjunctive traditional therapy contrast
Steunebrink et al., 2013 [36]	Randomized placebo-controlled trial	GTN patch plus eccentric training vs. placebo patch plus eccentric training	Secondary/adjunctive synthesis	Not pooled in main analysis; pharmacological adjunct over common exercise
Stasinopoulos and Stasinopoulos, 2004 [37]	RCT	Exercise programme vs. pulsed ultrasound vs. transverse friction	Secondary/narrative synthesis	Not pooled in main analysis; comparator heterogeneity
Liu et al., 2014 [38]	RCT	Low-level laser therapy vs. eccentric exercise vs. laser plus eccentric exercise	Secondary/adjunctive synthesis	Not pooled in main analysis; adjunctive and non-exercise arms require separate analysis
Blackwood and Ghazi, 2012 [39]	Pilot comparative clinical trial	Exercise programme vs. exercise plus transverse friction massage	Sensitivity/narrative candidate	Not pooled; pilot/manual therapy adjunct and limited extractable data
Rosety-Rodriguez et al., 2006 [40]	Comparative clinical study	Standard squat programme vs. eccentric decline-board programme	Historical/contextual evidence	Not pooled; historical design and incomplete compatible data
Jensen and Di Fabio, 1989 [41]	Experimental comparative study	Stretching alone vs. stretching plus isokinetic eccentric training	Historical/mechanistic evidence	Not pooled; historical/mechanistic and limited outcome comparability
Toustrup et al., 2025 [42]	Randomized crossover acute loading study	Single-session leg press at different repetition-maximum loads	Acute/mechanistic evidence	Not pooled; single-session physiological comparison
Holden et al., 2020 [43]	Randomized crossover acute study	Acute isometric vs. dynamic exercise exposure	Acute/mechanistic evidence	Not pooled; acute analgesic response only
Rio et al., 2015 [44]	Randomized crossover mechanistic study	Acute isometric vs. isotonic loading	Acute/mechanistic evidence	Not pooled; acute mechanistic response only
Pearson et al., 2020 [45]	Comparative longitudinal exercise study	Short-duration vs. long-duration isometric contractions	Sensitivity/narrative candidate	Not pooled; comparability limitations and no directly extractable numerical data after author contact
van Ark et al., 2013 [46]	Prospective case series	Exercise-based physical therapy after PRP	Narrative clinical evidence	Not pooled; no independent comparator

Note. RCT: randomized controlled trial; HSR: heavy slow resistance; PNE: percutaneous needle electrolysis; ESWT: extracorporeal shockwave therapy; USGET: ultrasound-guided galvanic electrolysis technique; GTN: glyceryl trinitrate; WBV: whole-body vibration; PRP: platelet-rich plasma. Studies marked as not pooled were retained for narrative, secondary, adjunctive, acute/mechanistic, or historical interpretation because they did not provide sufficiently comparable outcome data for the primary VISA-P/VISA final-score synthesis, involved a non-exercise comparator, had a non-randomized or acute design, or lacked extractable data despite author contact.

**Table 2:** Exercise loading and dose parameters among longitudinal clinical exercise interventions.

Study	Main Clinical Contrast	Loading/Dose Information Available from Report	Programme Duration/Follow-Up	Supervision/Adherence Information	Final Synthesis Status
Breda et al., 2021 [6]	PTLE vs. EET	PTLE used staged progression from isometric loading to isotonic, energy-storage and sport-specific loading; progression based on pain during single-leg squat (VAS <=3) and completion of at least 1 week at prior stage. EET used pain-provoking 25-degree decline-board eccentric squats twice daily for 12 weeks, with added backpack load when pain was minimal.	24-week primary endpoint; EET initial 12-week eccentric stage; return to sport allowed according to pain criteria.	Education and load-modification advice provided to both groups; adherence reported in original trial.	Included in primary VISA-P/VISA final-score meta-analysis
Agergaard et al., 2021 [7]	HSR vs. MSR	Both groups performed leg press and knee extension, 3 sessions/week. HSR progressed from 55% to 90% 1RM; MSR maintained 55% 1RM with matched volume. Sets progressed from 3 to 5; repetitions decreased over time.	12-week intervention; 52-week follow-up.	One weekly supervised session; remaining sessions unsupervised; training diary used; adherence 78% HSR and 86% MSR.	Included in primary VISA-P/VISA final-score meta-analysis
Agergaard et al., 2026 [9]	Short vs. extended restitution	Both groups used leg press and knee extension plus restricted impact activities. Load started at approximately 60% 1RM and progressed to approximately 75% 1RM. Short restitution trained 3 exercise days/week; extended restitution trained 1 exercise day/week.	12-week intervention; 12-week primary endpoint.	Supervised/monitored clinical trial; adherence and load restriction reported in trial.	Included in primary VISA-P/VISA final-score meta-analysis
Hjortshoej et al., 2025 [10]	LL-BFRT vs. HSRT	LL-BFRT: unilateral leg press and knee extension at 30% 1RM with 4 sets (30/15/15/15 reps) and cuff pressure progressed from 50% to 80% AOP. HSRT: same exercises, 4 sets with load progressing from 55% to 80% 1RM and decreasing repetitions.	12-week intervention; outcomes at 3, 6, 12 and 52 weeks.	One supervised session per week; electronic training diaries; attendance 77.3% LL-BFRT and 81.3% HSRT.	Included in primary VISA-P/VISA final-score meta-analysis
Kongsgaard et al., 2009 [11]	HSR vs. eccentric decline squat	ECC: 3 sets of 15 slow unilateral decline squats twice daily for 12 weeks. HSR: squat, leg press and hack squat, 3 sessions/week, 4 sets/exercise, progressing from 15RM to 6RM. Sports allowed if pain remained <=30/100 VAS.	12-week intervention; half-year follow-up.	One supervised session per week for ECC and HSR; adherence 89% ECC and 91% HSR; all exercise participants completed at least 75% sessions.	HSR vs. ECC included in primary meta-analysis; corticosteroid arm excluded from main exercise-loading synthesis
Jiang et al., 2024 [12]	Eccentric training + WBV 50 Hz vs. eccentric training alone	All groups performed quadriceps eccentric training for 8 weeks, 3 sessions/week. Vibration groups added 6 sets of WBV at 2 mm amplitude after eccentric training; frequency 30, 40 or 50 Hz. The 50 Hz arm was prespecified for main synthesis to avoid double counting.	8-week intervention.	No dropouts; 48/48 analyzed. Detailed adherence percentage NR. No adverse events reported.	50 Hz + eccentric vs. eccentric-alone contrast included in primary meta-analysis
Ruffino et al., 2021 [13]	Inertial flywheel resistance vs. HSR	Dose details partly available in report; numerical VISA-P data required derivation from figure and were not used in main extraction.	12-week intervention.	Not available in a directly extractable numerical format after author contact.	Sensitivity/narrative only
Jonsson and Alfredson, 2005 [14]	Eccentric vs. concentric quadriceps training	Decline-board eccentric training compared with concentric training; exact pooling data limited by unit-of-analysis and attrition concerns.	12-week intervention.	Compatible data were not available after author contact.	Sensitivity/narrative only
Stasinopoulos et al., 2012 [15]	Eccentric training plus static stretching vs. eccentric training alone	Eccentric training with or without static stretching; full compatible final-score extraction not available for primary synthesis.	4-week intervention; follow-up to 24 weeks.	Assignment and reporting limitations noted; compatible data were not available after author contact.	Secondary/sensitivity narrative only
van Ark et al., 2016 [16]	Isometric vs. isotonic exercise	Contraction-type contrast; outcomes reported over a short in-season programme with non-compatible summaries for main final-score synthesis.	4-week intervention.	Not available in a directly extractable numerical format after author contact.	Narrative/sensitivity only
Frohm et al., 2007 [17]	Eccentric overload device vs. decline eccentric	Device-based eccentric overload compared with decline-board eccentric training; pilot study with non-compatible summary statistics for main analysis.	12-week intervention.	Not available in a directly extractable numerical format after author contact.	Narrative/sensitivity only
Rieder et al., 2022 [18]	WBV vs. HSR vs. waitlist	Whole-body vibration and HSR compared with waitlist; outcome structure better suited to change-score or separate analysis rather than final-score active-loading synthesis.	Programme duration was not available in the extraction table or after author contact.	Not available in the report or after author contact.	Narrative or separate change-score analysis
Bahr et al., 2006 [19]	Eccentric decline-board training vs. open tenotomy	Exercise comparator involved decline-board eccentric training; surgical comparator prevents pooling with active exercise-loading comparisons.	12-week exercise phase; follow-up to 12 months.	Dose information was not available in the report or after author contact.	Separate exercise-vs.-surgery subgroup/narrative
Purdam et al., 2004 [20]	Standard single-leg squat vs. decline-board eccentric squat	Historical pilot comparison of squat angle/loading strategy; VISA/VISA-P not reported.	12-week programme.	Not available in the report or after author contact.	Narrative only; excluded from VISA-P synthesis
Fendri et al., 2026 [21]	Progressive eccentric tendon loading vs. usual activity continuation	Progressive eccentric programme described at study level; extractable final numerical data unavailable in current file set.	12 weeks.	No additional author data received.	Narrative only until extractable data become available
Magendran et al., 2025 [22]	Plyometric vs. aquatic training	Exercise modality contrast; dose and outcome comparability could not be established from the available records.	6 weeks.	No additional author data received.	Narrative candidate only
Xiao et al., 2024 [23]	HSR +/− high-dose therapeutic ultrasound	HSR arm available but randomized contrast primarily tests ultrasound adjunct.	Dose information was not available in the report or after author contact.	No additional author data received.	Secondary/adjunctive synthesis
Lee et al., 2020 [32]	Eccentric exercise +/− ESWT	Eccentric decline squat common to both arms; adjunctive ESWT contrast.	12 weeks.	Dose information was not available in the report or after author contact.	Secondary/adjunctive synthesis
Thijs et al., 2017 [33]	Eccentric training + ESWT/sham	Eccentric training common to both groups; ESWT vs. sham contrast.	Follow-up to 24 weeks.	Dose information was not available in the report or after author contact.	Secondary/adjunctive synthesis
Liu et al., 2014 [38]	Eccentric exercise, laser, or combined therapy	Exercise-only, laser-only and combined arms; grouping requires separate pre-specification.	4 weeks.	Not available in the report or after author contact.	Secondary/adjunctive synthesis
Blackwood and Ghazi, 2012 [39]	Exercise +/− transverse friction massage	Exercise programme with or without manual therapy adjunct.	3 weeks.	Not available in the report or after author contact.	Sensitivity/narrative only
Rosety-Rodriguez et al., 2006 [40]	Standard squat vs. decline squat programme	Historical comparative exercise study; dose/outcome compatibility could not be established from the available data.	Not available in the report or after author contact.	No additional author data received.	Historical/narrative only
Jensen and Di Fabio, 1989 [41]	Stretching vs. stretching + isokinetic eccentric training	Historical eccentric/isokinetic loading information; limited clinical outcome comparability.	8 weeks.	Not available in the report or after author contact.	Historical/mechanistic narrative only

Note. NR/no author response indicates that the information was not extractable from the available full-text report and was not supplied after author contact. These fields were not imputed or inferred. Primary pooling was restricted to longitudinal randomized exercise/loading comparisons with extractable VISA-P/VISA final-score data on the same 0–100 scale. Adjunctive contrasts, acute experiments, non-randomized designs, and studies with incomplete numerical data were retained for narrative or secondary synthesis. HSRT: heavy slow resistance training; LL-BFRT: low-load blood-flow restriction training; NR: not reported.

**Table 3:** Risk of bias for studies contributing to the primary VISA-P/VISA final-score meta-analysis.

Study	D1 Randomization	D2 Deviations	D3 Missing Data	D4 Measurement	D5 Reported Result	Overall
Breda 2021 [6]	Low risk	Some concerns	Some concerns	Some concerns	Low risk	Some concerns
Agergaard 2021 [7]	Some concerns	Some concerns	Some concerns	Some concerns	Low risk	Some concerns
Agergaard 2026 [9]	Low risk	Some concerns	Low risk	Some concerns	Low risk	Some concerns
Hjortshoej 2025 [10]	Low risk	Some concerns	Low risk	Some concerns	Low risk	Some concerns
Kongsgaard 2009 [11]	Some concerns	Some concerns	Some concerns	Some concerns	Some concerns	Some concerns
Jiang 2024 [12]	Some concerns	Some concerns	Low risk	Some concerns	Some concerns	Some concerns

Note. D1: bias arising from the randomization process; D2: bias due to deviations from intended interventions; D3: bias due to missing outcome data; D4: bias in measurement of the outcome; D5: bias in selection of the reported result. Assessments apply to VISA-P/VISA final score at the time point used in the primary synthesis.

**Table 4:** Primary comparative final-score synthesis dataset and study-level effects for VISA-P/VISA final score.

Study	Comparison	Weeks	*n* Arm 1/Arm 2	MD [95% CI]	Overall RoB 2
Breda 2021 [6]	PTLE vs. EET	24	38/38	9.10 [2.20, 16.00]	Some concerns
Agergaard 2021 [7]	HSR vs. MSR	12	21/21	−2.00 [−12.33, 8.33]	Some concerns
Agergaard 2026 [9]	Short vs. extended restitution	12	28/24	4.40 [−4.47, 13.27]	Some concerns
Hjortshoej 2025 [10]	LL-BFRT vs. HSRT	12	16/20	3.63 [−4.70, 11.96]	Some concerns
Kongsgaard 2009 [11]	HSR vs. ECC	12	13/12	3.00 [−6.93, 12.93]	Some concerns
Jiang 2024 [12]	Eccentric + WBV vs. eccentric	8	12/12	5.58 [2.23, 8.93]	Some concerns
Pooled effect	Random-effects REML + Hartung–Knapp	-	6 comparisons	5.16 [2.39, 7.93]	Some concerns body of evidence

**Table 5:** Summary of findings and GRADE certainty assessment for the primary outcome.

GRADE Domain	Assessment/Finding
Outcome	VISA-P/VISA final score
Body of evidence	6 RCTs/255 participants
Effect estimate	MD 5.16 points higher (95% CI 2.39 to 7.93)
Risk of bias	Serious
Inconsistency	Not serious
Indirectness	Serious
Imprecision	Not serious
Publication bias	Not formally assessed
Overall certainty	Low certainty
Importance	Critical

Note. Certainty started as high because the evidence came from randomized trials. The certainty was downgraded by one level for risk of bias because all studies contributing to the primary meta-analysis were judged as having some concerns in the outcome-specific RoB 2 assessment. The certainty was downgraded by one level for indirectness because the pooled estimate combined clinically distinct active exercise/loading comparisons rather than a single homogeneous intervention contrast. No downgrade was applied for inconsistency because statistical heterogeneity was negligible (I2 = 0.0%; tau2 = 0.000). No downgrade was applied for imprecision because the 95% CI excluded the null value, although the absolute effect was small. Publication bias could not be formally assessed because fewer than 10 comparisons were available; therefore, small-study effects remain an interpretive limitation.

## Data Availability

All data generated or analyzed during this study will be included in the article and its Supplementary Materials.

## Supplementary Materials

The following supporting information can be downloaded at: https://www.mdpi.com/article/10.3390/jfmk11030348/s1, Figure S1: Risk of bias assessment for studies included in the primary VISA-P/VISA meta-analysis; Figure S2: Domain-level summary of RoB 2 judgements across studies included in the primary VISA-P/VISA meta-analysis; Table S1: Full electronic search strategies; Table S2: Full-text reports excluded with PRISMA-compatible reasons; Table S3: Risk-of-bias and methodological appraisal of studies not included in the primary VISA-P/VISA meta-analysis; Table S4: GRADE evidence profile for the primary outcome; File S1: PRISMA 2020 Checklist.
