# Supplementary material for: Exercise Loading Strategies for Patellar Tendinopathy: A Systematic Review and Meta-Analysis of Clinical Outcomes
Source: J Funct Morphol Kinesiol. 2026 Aug 31;11(3):348. doi: 10.3390/jfmk11030348 (PMC13608764; doi:10.3390/jfmk11030348)
Supplement: Supplementary file 1 [file jfmk-11-00348-s001.zip › Supplementary Table S4 GRADE Primary Outcome.pdf]

Supplementary Table S4. GRADE evidence profile for the primary outcome

Purpose. This table documents the certainty-of-evidence judgment for the primary quantitative synthesis of VISA-P/VISA final score. Secondary outcomes were not assigned a pooled GRADE rating because they were not synthesized quantitatively in a sufficiently comparable manner.

Supplementary Table S4. GRADE evidence profile for the primary outcome

Outcome	No. of studies / design	No. of participants	Risk of bias	Inconsistency	Indirectness	Imprecision	Publication bias	Effect	Certainty	Importance
VISA-P/VISA final score at the end of the intervention or primary post-intervention time point	6 randomized controlled trials	255	Serious. All studies contributing to the primary meta-analysis were judged as some concerns. Main limitations were lack of participant blinding in exercise trials using a patient-reported outcome, incomplete reporting of allocation concealment in some studies, and incomplete prospective analysis transparency in older/smaller trials.	Not serious. Statistical heterogeneity was negligible (tau2 = 0.000; I2 = 0.0%; Q = 3.50; p = 0.624), and confidence intervals were broadly overlapping.	Serious. The synthesis pools several clinically distinct active loading contrasts rather than one unified intervention class: progressive tendon loading vs eccentric exercise, heavy vs moderate slow resistance, restitution/frequency contrast, low-load BFR vs HSR, HSR vs eccentric exercise, and eccentric exercise plus WBV vs eccentric exercise alone. Follow-up timing ranged from 8 to 24 weeks.	Not serious. The 95% CI excluded the null and was relatively narrow. The estimated effect is best interpreted as small; the CI does not support a large clinically important difference.	Not downgraded. Publication bias was not formally assessed because fewer than 10 comparisons contributed to the primary meta-analysis; therefore, small-study effects remain an interpretive limitation.	MD = 5.16 points (95% CI 2.39 to 7.93) on a 0-100 VISA-P/VISA scale, favouring the first-listed intervention.	Low (⊕⊕◯◯)	Critical

Note. The body of evidence started at high certainty because all studies contributing to the primary synthesis were randomized trials. The final rating was downgraded by one level for risk of bias and by one level for indirectness. MD: mean difference; CI: confidence interval; RCT: randomized controlled trial; VISA-P: Victorian Institute of Sport Assessment-Patella; BFR: blood-flow restriction; HSR: heavy slow resistance; WBV: whole-body vibration.
