# Supplementary material for: Exercise Loading Strategies for Patellar Tendinopathy: A Systematic Review and Meta-Analysis of Clinical Outcomes
Source: J Funct Morphol Kinesiol. 2026 Aug 31;11(3):348. doi: 10.3390/jfmk11030348 (PMC13608764; doi:10.3390/jfmk11030348)
Supplement: Supplementary file 1 [file jfmk-11-00348-s001.zip › Supplementary Table S3 NonPrimary RoB Appraisal.pdf]

Supplementary Table S3. Risk-of-bias and methodological appraisal of studies not included in the primary VISA-P/VISA meta-analysis

Purpose. This table documents how studies outside the primary final-score meta-analysis were handled for risk-of-bias and interpretive purposes. The assessment is role-specific: randomized longitudinal studies considered for sensitivity or secondary synthesis were appraised using RoB 2 principles; non-randomized, quasi-randomized, historical, or comparative clinical studies were classified using a ROBINS-I-oriented methodological appraisal; acute or mechanistic studies were not rated as longitudinal clinical effectiveness trials. No unavailable data were imputed, and NR/no author response was treated as a reporting limitation rather than as recoverable evidence.

Study	Design	Contrast / role	Assessment approach	RoB / methodological concern	Main rationale	Final use
Ruffino et al., 2021	RCT	Flywheel resistance vs HSR	RoB 2	Some concerns	Outcome data required derivation from figure for the review dataset; participant blinding not feasible; reporting constraints limit confidence in precise effect extraction.	Sensitivity/narrative only unless extractable numerical data are verified.
Jonsson and Alfredson, 2005	Prospective randomized trial	Eccentric vs concentric training	RoB 2	High risk / serious concerns for quantitative synthesis	Small trial; unit-of-analysis concerns related to tendons rather than independent participants; differential dropout and limited reporting of allocation/missing-data handling.	Narrative or sensitivity only; not included in primary pooled estimate.
Stasinopoulos et al., 2012	Controlled clinical trial / quasi-randomized features	Eccentric + stretching vs eccentric alone	ROBINS-I-oriented appraisal	Serious risk	Allocation by alternation or non-random features; estimates reported as mean with 95% CI rather than directly extractable mean/SD; design not aligned with primary RCT synthesis.	Narrative or separate sensitivity only.
van Ark et al., 2016	RCT	Isometric vs isotonic exercise	RoB 2	Some concerns	Short follow-up; outcome reporting used median/IQR for relevant data; non-blinding expected for exercise interventions; limited compatibility with final-score MD pooling.	Narrative/sensitivity; not included in primary final-score meta-analysis.

Frohm et al., 2007	Pilot RCT	Eccentric overload device vs decline-board eccentric training	RoB 2	Some concerns	Pilot design and small sample; relevant outcome information not fully compatible with primary final-score extraction; limited precision.	Narrative/sensitivity only.
Rieder et al., 2022	Randomized equivalence trial	WBV vs HSR vs waitlist	RoB 2	Some concerns	Clinically relevant randomized evidence but primarily compatible with change-score or separate contrast synthesis rather than the final-score active-comparison dataset.	Separate synthesis/narrative; not pooled with primary final-score analysis.
Bahr et al., 2006	RCT	Eccentric decline-board training vs surgery	RoB 2	Some concerns	Comparator was surgical treatment rather than another active exercise-loading strategy; participant blinding not feasible; clinical question distinct from the primary synthesis.	Separate exercise-versus-surgery narrative only.
Purdam et al., 2004	Pilot comparative study	Standard single-leg squat vs decline-board eccentric squat	ROBINS-I-oriented appraisal	Serious risk	Pilot/historical comparative evidence; not all primary review outcomes reported, and VISA-P was not available for the primary outcome dataset.	Narrative context for loading-angle rationale; not eligible for VISA-P meta-analysis.
Ferreira et al., 2025	RCT	Dynamic Tape + PTLE vs sham tape + exercise	RoB 2 if pooled secondarily	Not included in primary RoB set	Adjunctive tape contrast over an exercise base; does not isolate exercise-loading strategy.	Secondary/adjunctive synthesis only.
Xiao et al., 2024	RCT	HSR plus high-dose therapeutic ultrasound vs HSR vs ultrasound	RoB 2 if pooled secondarily	Not included in primary RoB set	Adjunctive ultrasound contrast; dose details and extractability require separate verification.	Secondary/adjunctive synthesis only.
Zhao et al., 2022	RCT	Baduanjin training plus shockwave vs shockwave alone	RoB 2 if pooled secondarily	Not included in primary RoB set	Mixed exercise-adjunct intervention; clinical contrast not comparable with active loading-strategy comparisons.	Secondary/narrative only.
Sanchez-Gomez et al., 2022	Pilot RCT	HMB supplementation + rehabilitation vs placebo + rehabilitation	RoB 2 if pooled secondarily	Not included in primary RoB set	Supplementation adjunct over rehabilitation; does not estimate loading-strategy contrast.	Secondary/narrative only.

Sharif et al., 2022	RCT	Dry needling + conventional physical therapy vs conventional physical therapy	RoB 2 if pooled secondarily	Not included in primary RoB set	Adjunctive dry needling contrast over common therapy base.	Secondary/adjunctive synthesis only.
Stepanenko et al., 2022	Randomized clinical study	Exercise rehabilitation + laser vs exercise rehabilitation alone	RoB 2 if pooled secondarily	Not included in primary RoB set	Adjunctive laser contrast over exercise base.	Secondary/adjunctive synthesis only.
Lopez-Royo et al., 2021	RCT	Dry needling/PNE/sham needling plus eccentric exercise	RoB 2 if pooled secondarily	Not included in primary RoB set	Needling adjunct contrast; exercise was common or not the isolated randomized loading variable.	Secondary/adjunctive synthesis only.
Olesen et al., 2021	Randomized placebo-controlled trial	IGF-1 injection + HSR vs saline + HSR	RoB 2 if pooled secondarily	Not included in primary RoB set	Injection adjunct over common HSR base.	Secondary/adjunctive synthesis only.
Lee et al., 2020	RCT	Eccentric decline squat vs eccentric + ESWT	RoB 2 if pooled secondarily	Not included in primary RoB set	Adjunctive ESWT contrast; not a pure exercise-loading comparison.	Secondary/narrative only.
Thijs et al., 2017	Randomized placebo-controlled trial	ESWT + eccentric training vs sham ESWT + eccentric training	RoB 2 if pooled secondarily	Not included in primary RoB set	Adjunctive ESWT over common eccentric exercise base.	Secondary/adjunctive synthesis only.
Abat et al., 2016	RCT	USGET + eccentric exercise vs electrophysiotherapy + eccentric exercise	RoB 2 if pooled secondarily	Not included in primary RoB set	Adjunctive invasive/electrotherapy contrast; exercise not isolated as randomized loading strategy.	Secondary/narrative only.
Steunebrink et al., 2013	Randomized placebo-controlled trial	GTN patch + eccentric training vs placebo patch + eccentric training	RoB 2 if pooled secondarily	Not included in primary RoB set	Pharmacologic adjunct over common exercise base.	Secondary/adjunctive synthesis only.
Liu et al., 2014	RCT	Laser, eccentric exercise, and combined arms	RoB 2 if pooled secondarily	Not included in primary RoB set	Adjunctive modality with multiple arms; grouping would require prespecified secondary analysis.	Secondary/narrative only.
Toustrup et al., 2025	Randomized crossover acute loading study	Single-session leg press loads	Not rated as longitudinal effectiveness trial	Not applicable for primary RoB set	Acute/mechanistic load-response study; no longitudinal rehabilitation effect.	Mechanistic context only.
Holden et al., 2020	Randomized crossover acute study	Acute isometric vs dynamic exposure	Not rated as longitudinal effectiveness trial	Not applicable for primary RoB set	Immediate analgesic/exposure study; not a treatment programme.	Mechanistic context only.
Rio et al., 2015	Randomized crossover mechanistic study	Acute isometric vs isotonic loading	Not rated as longitudinal effectiveness trial	Not applicable for primary RoB set	Immediate analgesic and neurophysiological response; not longitudinal clinical effectiveness.	Mechanistic context only.

Pearson et al., 2020	Comparative longitudinal exercise study	Short- vs long-duration isometrics	ROBINS-I-oriented appraisal if clinically synthesized	Serious concerns / limited causal inference	Comparability and reporting require caution; retained for duration-response context rather than primary effectiveness pooling.	Sensitivity/narrative only.
Blackwood and Ghazi, 2012	Pilot comparative clinical trial	Exercise vs exercise + transverse friction massage	ROBINS-I-oriented appraisal	Serious concerns	Pilot design and adjunctive manual therapy contrast; limited precision and reporting.	Sensitivity/narrative only.
Rosety-Rodriguez et al., 2006	Comparative clinical study	Standard squat programme vs decline-board programme	ROBINS-I-oriented appraisal	Serious concerns	Historical comparative design with limited reporting and uncertain allocation methods.	Historical/sensitivity context only.
Jensen and Di Fabio, 1989	Experimental comparative study	Stretching vs stretching + isokinetic eccentric training	ROBINS-I-oriented appraisal	Serious concerns	Historical intervention study with limited outcome comparability to VISA-P-era evidence.	Historical/mechanistic context only.

Note. These judgements do not replace the outcome-specific RoB 2 assessment for the six studies included in the primary meta-analysis. They are intended to prevent inappropriate mixing of clinically distinct evidence streams and to support transparent interpretation of sensitivity, adjunctive, historical, and mechanistic evidence.
