# Supplementary material for: Exercise Loading Strategies for Patellar Tendinopathy: A Systematic Review and Meta-Analysis of Clinical Outcomes
Source: J Funct Morphol Kinesiol. 2026 Aug 31;11(3):348. doi: 10.3390/jfmk11030348 (PMC13608764; doi:10.3390/jfmk11030348)
Supplement: Supplementary file 1 [file jfmk-11-00348-s001.zip › Supplementary Table S2 Full Text Exclusions.pdf]

Supplementary Table S2. Full-text reports excluded with PRISMA-compatible reasons

Purpose. This table documents the 56 full-text reports excluded after eligibility assessment. It supports the PRISMA 2020 flow diagram and provides transparent, auditable exclusion reasons. Reports that could not be retrieved are not included here and should be reported separately as reports not retrieved.

Summary by broad reason category. Duplicate/overlapping publication or secondary analysis: 13; wrong intervention/no eligible therapeutic exercise intervention: 19; wrong population/condition or prevention-risk study: 13; wrong study design/aim or non-original research: 8; retracted article: 1; conference abstract only: 1; wrong publication type: 1. Total full-text reports excluded: 56.

Primary PRISMA-compatible reason	n
Duplicate/overlapping publication or secondary analysis	13
Wrong intervention/no eligible therapeutic exercise intervention	19
Wrong population/condition or prevention-risk study	13
Wrong study design/aim or non-original research	8
Retracted article	1
Conference abstract only	1
Wrong publication type	1
Total full-text reports excluded	56

No.	Author / year	Report title / short description	Assessment status	Primary standardized exclusion reason	Eligibility justification / audit note
8	López-Royo et al., 2024	Functionality and jump performance following treatment interventions for patellar tendinopathy	Full text assessed	Duplicate/overlapping publication / secondary analysis	Secondary report from the same trial represented by López-Royo et al., 2021. Provides complementary functional/jump outcomes but no independent clinical effectiveness dataset for the review.
9	Doménech-García et al., 2024	Placebo and nocebo effects in patellar tendinopathy	Full text assessed	Duplicate/overlapping publication / secondary analysis	Secondary analysis focused on placebo/nocebo effects and pain related to needling, overlapping with the primary López-Royo et al. randomized trial.
10	Sharif et al., 2023	Effectiveness of ultrasound-guided dry needling as an adjunct to conventional physical therapy	Full text assessed	Retracted article	The publication was clearly marked as retracted. Retracted reports were not eligible for the review or meta-analysis.
11	Fernández-Sanchis et al., 2022	A comparative study of treatment interventions for patellar tendinopathy: a secondary cost-effectiveness analysis	Full text assessed	Duplicate/overlapping publication / secondary cost-effectiveness analysis	Economic evaluation derived from the López-Royo et al. randomized trial. Outcomes focused on costs, QALYs and cost-effectiveness rather than independent clinical effectiveness.
13	Breda et al., 2022	Decreasing patellar tendon stiffness during exercise therapy	Full text assessed	Duplicate/overlapping publication / secondary mechanistic analysis	Companion mechanistic report from the JUMPER trial, already represented by the primary clinical trial of Breda et al., 2021.
16	Barker-Davies et al., 2022	High-volume image-guided injections in Achilles and patellar tendinopathy	Full text assessed	Wrong intervention	Randomized intervention was high-volume image-guided injection with/without steroid, not exercise therapy. Population also combined Achilles and patellar tendinopathy without an independently extractable exercise-loading contrast.
26	van Ark et al., 2018	Clinical improvements are not explained by changes in tendon structure	Full text assessed	Duplicate/overlapping publication / secondary structural analysis	Secondary structural report linked to the van Ark et al. exercise trial. Did not provide an independent therapeutic comparison for the review.
27	Esculier et al., 2018	Predictors of clinical success in runners with patellofemoral pain	Full text assessed	Wrong population	Population was runners with patellofemoral pain rather than adults with patellar tendinopathy.
28	Ramskov et al., 2018	Progression in running intensity or running volume and running-related injuries	Full text assessed	Wrong population / prevention-risk study	Study evaluated injury risk in healthy/recreational runners, not treatment of established patellar tendinopathy.
31	Gual et al., 2016	In-season inertial resistance training with eccentric overload in a sports population at risk	Full text assessed	Wrong population / prevention study	Participants were a sports population at risk rather than patients with diagnosed patellar tendinopathy; the study was preventive/performance-oriented.
35	Rigby et al., 2015	Wireless versus wired iontophoresis for treating patellar tendinopathy	Full text assessed	Wrong intervention	Trial evaluated dexamethasone iontophoresis devices/sham and did not involve a therapeutic tendon-loading exercise intervention.

No.	Author / year	Report title / short description	Assessment status	Primary standardized exclusion reason	Eligibility justification / audit note
38	Willberg et al., 2011	Sclerosing polidocanol injections or arthroscopic shaving for patellar tendinopathy	Full text assessed	Wrong intervention	Intervention compared sclerosing injections with arthroscopic shaving; no eligible exercise-loading intervention was evaluated.
42	Deng et al., 2026	Prognostic value of ultrasound and MRI features	Full text assessed	Duplicate/overlapping publication / secondary prognostic analysis	Secondary prognostic analysis derived from an exercise trial; not an independent clinical effectiveness study.
43	Fang et al., 2026	UTE-MRI radiomics as a predictor of clinical outcomes	Full text assessed	Duplicate/overlapping publication / secondary prognostic-imaging analysis	Radiomics/prognostic analysis using data from an existing exercise cohort/trial; no independent intervention comparison.
44	Pengked et al., 2025	Badminton-related musculoskeletal injuries in senior players	Full text assessed	Wrong study design / wrong intervention	Epidemiological/cross-sectional injury study; did not evaluate exercise therapy for patellar tendinopathy.
45	Ruffino et al., 2025	Beliefs and experiences following a rehabilitation programme for patellar tendinopathy	Full text assessed	Duplicate/overlapping publication / nested qualitative study	Qualitative companion study nested within the Ruffino et al. rehabilitation trial; useful context but not an independent effectiveness report.
46	Deng et al., 2025	Do physical or imaging changes explain the effectiveness of progressive tendon-loading exercises?	Full text assessed	Duplicate/overlapping publication / secondary mediation analysis	Mediation analysis of the JUMPER trial; did not constitute a separate therapeutic comparison.
48	Deng et al., 2025	Long-term prognosis of athletes with patellar tendinopathy receiving physical therapy	Full text assessed	Duplicate/overlapping publication / long-term follow-up not independent	Long-term follow-up of participants from a prior exercise trial; did not provide an independent randomized comparison for primary synthesis.
49	Svensson et al., 2024	Application of multispectral optoacoustic tomography in musculoskeletal disorders	Full text assessed	Wrong intervention / wrong study aim	Imaging feasibility/pilot study; no exercise intervention or clinical effectiveness comparison.
51	Breda & Oei, 2023	MRI of tendinopathy using ultrashort TE sequences	Full text assessed	Wrong publication type / book chapter	Book chapter on UTE-MRI techniques and applications. Not a primary clinical intervention study and not eligible for meta-analysis.
52	Deng et al., 2023	Association between physical tests and patient-reported outcomes	Full text assessed	Duplicate/overlapping publication / secondary prognostic analysis	Secondary analysis from the JUMPER study; examined prognostic/associative questions rather than an independent intervention effect.
53	Park et al., 2022	Persons with patellar tendinopathy exhibit anatomical features	Full text assessed	Wrong intervention / wrong study design	Comparative anatomical/biomechanical study; no therapeutic exercise intervention was evaluated.
54	Breda et al., 2021	Association between UTE-MRI T2* relaxation times and symptoms during exercise therapy	Full text assessed	Duplicate/overlapping publication / secondary imaging analysis	Imaging companion analysis within the JUMPER trial; not counted as an independent clinical effectiveness study.
55	Agergaard et al., 2021	Mechanical properties and UTE-T2* in patellar tendinopathy	Full text assessed	Duplicate/overlapping publication / secondary mechanistic-imaging analysis	Mechanistic/imaging report linked to the Agergaard load trial; did not provide an independent clinical comparison.
58	Wiesinger et al., 2020	Mechanical and material tendon properties in proximal patellar tendinopathy	Full text assessed	Wrong intervention / wrong study design	Cross-sectional comparison of tendon properties; no therapeutic intervention.
59	Tamura et al., 2020	Effect of Kinesio-tape on pain and vertical jump performance	Full text assessed	Wrong intervention	Evaluated taping effects during functional tasks; no therapeutic exercise-loading programme was tested.
61	Rosen et al., 2018	Relationship between acute pain and dynamic postural stability indices	Full text assessed	Wrong intervention / wrong study design	Observational/exploratory study on pain and postural stability; no intervention.
62	Lang et al., 2017	Arthroscopic patellar release for chronic symptomatic patellar tendinopathy	Full text assessed	Wrong intervention	Surgical cohort/intervention; no eligible exercise comparator for the primary review question.
63	de Vries et al., 2015	Effect of patellar strap and sports tape on pain in patellar tendinopathy	Full text assessed	Wrong intervention	Trial evaluated strap/tape effects, not therapeutic exercise or tendon-loading rehabilitation.
64	Rio et al., 2015	Elevated corticospinal excitability in patellar tendinopathy	Full text assessed	Wrong intervention / wrong study design	Cross-sectional neurophysiological study; no therapeutic intervention.
65	Silva et al., 2016	Lower limb strength and flexibility in athletes with and without patellar tendinopathy	Full text assessed	Wrong intervention / wrong study design	Observational comparison of strength/flexibility; no treatment intervention.
66	Wearing et al., 2015	Tendinopathy alters ultrasound transmission in the patellar tendon during squatting	Full text assessed	Wrong intervention / wrong study design	Biomechanical/ultrasonographic study during squatting; no clinical exercise intervention.
67	Morton et al., 2014	Equivalence of online and clinician administration of a questionnaire	Full text assessed	Wrong study aim	Methodological validation/equivalence study for questionnaire administration; not an intervention study.

No.	Author / year	Report title / short description	Assessment status	Primary standardized exclusion reason	Eligibility justification / audit note
68	Janssen et al., 2014/2015	Patellar tendinopathy risk factors differ between elite and sub-elite volleyball players	Full text assessed	Wrong population / wrong intervention	Risk-factor/biomechanical study in volleyball players without a therapeutic exercise intervention.
69	Edwards et al., 2014	Alterations to landing technique and patellar tendon loading in response to fatigue	Full text assessed	Wrong population / wrong intervention	Biomechanical fatigue study in asymptomatic athletes; no patellar tendinopathy treatment.
70	Smith & Sellon, 2014	Comparing PRP injections with ESWT for chronic patellar tendinopathy	Full text assessed	Not original research / secondary commentary	Journal club/commentary on another trial; not a primary report.
72	Nielsen et al., 2014	Excessive progression in weekly running distance and risk of running-related injuries	Full text assessed	Wrong population / prevention-risk study	Prospective injury-risk study in novice runners; did not treat diagnosed patellar tendinopathy.
73	Rio et al., 2014	Exercise affects cortical inhibition and reduces pain in patellar tendinopathy	Full text assessed	Conference abstract only	Conference abstract without sufficient full-text methodological and outcome data; not eligible as a full-text report.
75	Pruna et al., 2013	Tendinopatía rotuliana. Modelo de actuación terapéutica en el deporte	Full text assessed	Wrong study design / narrative therapeutic model	Narrative/therapeutic model article; not a primary intervention study.
77	Edwards et al., 2010/2012	Characterizing patellar tendon loading during the landing phases of a stop-jump task	Full text assessed	Wrong population / wrong intervention	Biomechanical loading study in asymptomatic participants; no therapeutic intervention.
79	Ackermann & Renström, 2012	Tendinopathy in sport	Full text assessed	Wrong study design / narrative review	Narrative clinical review; no primary data.
80	Hernández-Sánchez et al., 2011	Cross-cultural adaptation of the VISA-P score for the Spanish population	Full text assessed	Wrong study aim / measurement validation study	Psychometric/cross-cultural validation of VISA-P; not an intervention study.
81	Malliaras et al., 2010	Temporal sequence of greyscale ultrasound changes and pain	Full text assessed	Wrong intervention / observational imaging study	Longitudinal observational imaging study; no therapeutic intervention.
82	Kraemer & Knobloch, 2009	Soccer-specific balance training for hamstring muscle and patellar/Achilles tendon injuries	Full text assessed	Wrong population / prevention study	Preventive programme in football players; did not treat established patellar tendinopathy.
84	Cumps et al., 2008	Preventive intervention programme on anterior knee pain in volleyball players	Full text assessed	Wrong population / prevention study	Prevention study for anterior knee pain in volleyball players; patellar tendinopathy was not the treated clinical condition.
85	Hyman, 2008	Jumper's knee in volleyball athletes: advancements in diagnosis and treatment	Full text assessed	Wrong study design / narrative review	Narrative update; not original intervention research.
86	Bisseling et al., 2007	Relationship between landing strategy and patellar tendinopathy in volleyball	Full text assessed	Wrong intervention / observational biomechanical study	Observational biomechanical study; no therapeutic exercise intervention.
87	Joy, 2007	Predicting lower extremity stress fractures in young women recruited into the Marine Corps	Full text assessed	Wrong population / wrong condition	Article was unrelated to patellar tendinopathy treatment and concerned stress fracture prediction.
88	Kongsgaard et al., 2006	Decline eccentric squats increase patellar tendon loading compared with standard eccentric squats	Full text assessed	Wrong population / biomechanical mechanistic study	Mechanistic biomechanical study in healthy participants; informative for rationale but not eligible as treatment evidence.
90	Alfredson et al., 2006	Sclerosing polidocanol injections for chronic painful shoulder impingement syndrome	Full text assessed	Wrong population / wrong anatomical condition	Study concerned shoulder/supraspinatus condition, not patellar tendinopathy.
94	Gaida et al., 2004	Unilateral and bilateral patellar tendinopathy: anthropometry, body composition, muscle strength	Full text assessed	Wrong intervention / observational risk-factor study	Observational comparison of athlete characteristics; no therapeutic intervention.
95	Lian et al., 2003	Performance characteristics of volleyball players with patellar tendinopathy	Full text assessed	Wrong intervention / observational performance-risk study	Performance/risk-factor study; no treatment intervention.
97	Schmid et al., 2002	Is impingement the cause of jumper's knee? Dynamic and static MRI	Full text assessed	Wrong intervention / diagnostic-mechanistic imaging study	Diagnostic/mechanistic MRI study; no therapeutic exercise intervention.
98	Shrier & Garrick, 2001	Leg exercises reduce jumper's knee pain	Full text assessed	Not original research / secondary summary	Secondary summary/commentary of another primary study; not counted independently.
99	Lian et al., 1996	Characteristics of the leg extensors in male volleyball players with jumper's knee	Full text assessed	Wrong intervention / observational performance-risk study	Observational comparison of leg extensor characteristics; no intervention.

No.	Author / year	Report title / short description	Assessment status	Primary standardized exclusion reason	Eligibility justification / audit note
100	Rubinstein et al., 1994	Isolated autogenous bone-patellar tendon-bone graft site morbidity	Full text assessed	Wrong population / wrong intervention	Study concerned donor-site morbidity after ACL graft harvest, not treatment of primary patellar tendinopathy.

Note. The five reports not retrieved after targeted retrieval attempts were not assessed for eligibility and are therefore not listed as full-text exclusions. They should remain in the PRISMA flow diagram under reports not retrieved. One primary standardized exclusion reason was assigned to each excluded full-text report to avoid double counting.

CENTRAL/Ovid search note. The 11 unique completed journal-article records identified after CENTRAL/Ovid deduplication were excluded at title/abstract screening. They did not undergo full-text assessment and are therefore not listed as full-text exclusions in this table.
