# Supplementary material for: Exercise Loading Strategies for Patellar Tendinopathy: A Systematic Review and Meta-Analysis of Clinical Outcomes
Source: J Funct Morphol Kinesiol. 2026 Aug 31;11(3):348. doi: 10.3390/jfmk11030348 (PMC13608764; doi:10.3390/jfmk11030348)
Supplement: Supplementary file 1 [file jfmk-11-00348-s001.zip › Supplementary Table S1. Full electronic search strategies.pdf]

Supplementary Table S1. Full electronic search strategies

Project: Exercise Loading Strategies for Patellar Tendinopathy: A Systematic Review and Meta-Analysis of Clinical Outcomes
Note. The active electronic search sources were MEDLINE/PubMed, Scopus, Web of Science Core Collection, SPORTDiscus, and CENTRAL via Ovid/EBMR Reviews. MEDLINE/PubMed, Scopus, Web of Science Core Collection, and SPORTDiscus were searched on 9 April 2026. CENTRAL via Ovid/EBMR Reviews was searched on 01 May 2026. Embase was not searched because reproducible access/export could not be implemented within the available review infrastructure. No date restriction was applied at the search stage where specified. For CENTRAL/Ovid, the search retrieved 242 records; 127 completed journal-article records were exported for deduplication and title/abstract screening, while 115 records outside this predefined completed journal-article screening subset were documented as removed before screening because they were not direct inputs to the completed published clinical-study screening set.

**Database / source**	**Platform / interface**	**Date searched**	**Operational limits / filters**	**Records retrieved**
MEDLINE/PubMed	PubMed	9 April 2026	No date restriction; NOT (animals[mh] NOT humans[mh]); trial-oriented search terms included in string	178
Scopus	Elsevier Scopus Advanced Search	9 April 2026	DOCTYPE(ar) and SRCTYPE(j)	184
Web of Science Core Collection	Clarivate Web of Science; Topic field or Advanced Search / Query Builder	9 April 2026	Advanced Search version uses DT=(Article)	221
SPORTDiscus	EBSCOhost Advanced Search	9 April 2026	Three search rows connected with AND; peer-reviewed / journal article filters recommended as available	117
CENTRAL	Ovid / EBMR Reviews - Cochrane Central Register of Controlled Trials	01 May 2026	No date restriction; final search line 5 AND 8; completed journal-article subset exported for deduplication and title/abstract screening (n = 127)	242

Total records retrieved before deduplication: 942 (700 from MEDLINE/PubMed, Scopus, Web of Science Core Collection, and SPORTDiscus plus 242 from CENTRAL/Ovid). For CENTRAL/Ovid, 127 completed journal-article records were exported for deduplication and title/abstract screening; 115 records outside this predefined completed journal-article screening subset were documented as removed before screening; 11 CENTRAL/Ovid journal-article records were unique after deduplication and all were excluded at title/abstract screening.

MEDLINE via PubMed
Records retrieved: 178
Operational instructions / limits: Use as a single search string in PubMed. No date restriction applied at the search stage.

((("Patellar Ligament"[Mesh] AND "Tendinopathy"[Mesh]) OR "patellar tendinopathy"[tiab] OR "patellar tendinopathies"[tiab] OR "patellar tendinosis"[tiab] OR "patellar tendonitis"[tiab] OR "patellar tendinitis"[tiab] OR "patella tendinopathy"[tiab] OR "patella tendinosis"[tiab] OR "patella tendonitis"[tiab] OR "patella tendinitis"[tiab] OR "jumper's knee"[tiab] OR "jumpers knee"[tiab]) AND ("Exercise Therapy"[Mesh] OR "Exercise"[Mesh] OR "Resistance Training"[Mesh] OR "Blood Flow Restriction Therapy"[Mesh] OR exercis*[tiab] OR "exercise therapy"[tiab] OR "exercise therapies"[tiab] OR "exercise program*"[tiab] OR "training program*"[tiab] OR "resistance training"[tiab] OR "strength training"[tiab] OR "heavy slow resistance"[tiab] OR "heavy slow resistance training"[tiab] OR eccentric*[tiab] OR isometric*[tiab] OR isotonic*[tiab] OR "blood flow restriction"[tiab] OR "blood-flow restriction"[tiab] OR BFR[tiab] OR BFRT[tiab] OR "progressive tendon loading"[tiab] OR "progressive tendon-loading"[tiab] OR "tendon loading"[tiab] OR "decline squat*"[tiab] OR "single-leg decline squat*"[tiab]) AND (randomized controlled trial[pt] OR controlled clinical trial[pt] OR random*[tiab] OR trial[tiab] OR placebo[tiab] OR sham[tiab] OR groups[tiab])) NOT (animals[mh] NOT humans[mh])

Scopus
Records retrieved: 184
Operational instructions / limits: Use in Advanced Search. This version already restricts results to articles and journals.

TITLE-ABS-KEY(("patellar tendinopathy" OR "patellar tendinopathies" OR "patellar tendinosis" OR "patellar tendonitis" OR "patellar tendinitis" OR "patella tendinopathy" OR "patella tendinosis" OR "patella tendonitis" OR "patella tendinitis" OR "jumper's knee" OR "jumpers knee") AND (exercis* OR "exercise therapy" OR "exercise therapies" OR "exercise program*" OR "training program*" OR "resistance training" OR "strength training" OR "heavy slow resistance" OR "heavy slow resistance training" OR eccentric* OR isometric* OR isotonic* OR "blood flow restriction" OR "blood-flow restriction" OR BFR OR BFRT OR "progressive tendon loading" OR "progressive tendon-loading" OR "tendon loading" OR "decline squat*" OR "single-leg decline squat*") AND (random* OR trial OR placebo OR sham OR "controlled trial")) AND DOCTYPE(ar) AND SRCTYPE(j)

Web of Science Core Collection
Records retrieved: 221
Operational instructions / limits: Recommended operational version for Fielded Search with the field set to Topic. Do not add TS= in the search box when using the Topic field selector.

3.1 Fielded Search / Topic
("patellar tendinopathy" OR "patellar tendinopathies" OR "patellar tendinosis" OR "patellar tendonitis" OR "patellar tendinitis"OR "patella tendinopathy" OR "patella tendinosis" OR "patella tendonitis" OR "patella tendinitis" OR "jumper's knee" OR "jumpers knee") AND (exercis* OR "exercise therapy" OR "exercise therapies" OR "exercise program*"OR "training program*" OR "resistance training" OR "strength training" OR "heavy slow resistance" OR "heavy slow resistance training" OR eccentric* OR isometric* OR isotonic* OR "blood flow restriction" OR "blood-flow restriction" OR BFR OR BFRT OR "progressive tendon loading" OR "progressive tendon-loading" OR "tendon loading" OR "decline squat*" OR "single-leg decline squat*") AND (random* OR trial OR placebo OR sham OR "controlled trial")

Alternative version for Advanced Search / Query Builder:
3.2 Advanced Search / Query Builder
TS=(("patellar tendinopathy" OR "patellar tendinopathies" OR "patellar tendinosis" OR "patellar tendonitis" OR "patellar tendinitis" OR "patella tendinopathy" OR "patella tendinosis" OR "patella tendonitis" OR "patella tendinitis" OR "jumper's knee" OR "jumpers knee") AND (exercis* OR "exercise therapy" OR "exercise therapies" OR "exercise program*" OR "training program*" OR "resistance training" OR "strength training" OR "heavy slow resistance" OR "heavy slow resistance training" OR eccentric* OR isometric* OR isotonic* OR "blood flow

restriction" OR "blood-flow restriction" OR BFR OR BFRT OR "progressive tendon loading" OR "progressive tendon-loading" OR "tendon loading" OR "decline squat*" OR "single-leg decline squat*") AND (random* OR trial OR placebo OR sham OR "controlled trial")) AND DT=(Article)

SPORTDiscus via EBSCOhost
Records retrieved: 117

Operational instructions / limits: Apply as three separate rows in Advanced Search, connected with AND. Keep the field as “All fields” in each row. Do not write AB, S1, S2, or S3 inside the search boxes.

4.1 Row 1 – Condition

("patellar tendinopathy" OR "patellar tendinopathies" OR "patellar tendinosis" OR "patellar tendonitis" OR "patellar tendinitis" OR "patella tendinopathy" OR "patella tendinosis" OR "patella tendonitis" OR "patella tendinitis" OR "jumper's knee" OR "jumpers knee")

4.2 Row 2 – Intervention

(exercis* OR "exercise therapy" OR "exercise therapies" OR "exercise program*" OR "training program*" OR "resistance training" OR "strength training" OR "heavy slow resistance" OR "heavy slow resistance training" OR eccentric* OR isometric* OR isotonic* OR "blood flow restriction" OR "blood-flow restriction" OR BFR OR BFRT OR "progressive tendon loading" OR "progressive tendon-loading" OR "tendon loading" OR "decline squat*" OR "single-leg decline squat*")

4.3 Row 3 – Trial filter

(random* OR trial OR placebo OR sham OR "controlled trial")

Recommended filters after running the search: peer-reviewed / journal article, as available in the interface.

Abbreviations and operational notes. BFR: blood flow restriction; BFRT: blood-flow restriction training; MeSH: Medical Subject Headings; TS: Topic Search. The strings above preserve the operational search record supplied for manuscript and PRISMA documentation.

CENTRAL via Ovid/EBMR Reviews

Records retrieved: 242
Operational instructions / limits: Run in Ovid Advanced Search within EBMR Reviews - Cochrane Central Register of Controlled Trials. No date restriction was applied. The subject heading Blood Flow Restriction Therapy was accepted by Ovid. The final line was 5 AND 8. After retrieval of 242 records, the completed journal-article subset (n = 127) was exported for deduplication against the master set and title/abstract screening. Eleven records were unique after deduplication, and all were excluded at title/abstract screening; none required full-text retrieval.

1. exp Patellar Ligament/
2. exp Tendinopathy/
3. 1 AND 2
4. ("patellar tendinopathy" or "patellar tendinopathies" or "patellar tendinosis" or "patellar tendonitis" or "patellar tendinitis" or "patella tendinopathy" or "patella tendinosis" or "patella tendonitis" or "patella tendinitis" or "jumper's knee" or "jumpers knee").mp.
5. 3 OR 4
6. exp Exercise Therapy/ or exp Exercise/ or exp Resistance Training/ or exp Blood Flow Restriction Therapy/
7. (exercis* or "exercise therapy" or "exercise therapies" or "exercise program*" or "training program*" or "resistance training" or "strength training" or "heavy slow resistance" or "heavy slow resistance training" or eccentric* or isometric* or isotonic* or "blood flow restriction" or "blood-flow restriction" or BFR or BFRT or "progressive tendon loading" or "progressive tendon-loading" or "tendon loading" or "decline squat*" or "single-leg decline squat*" or squat* or "leg press" or "knee extension" or "whole body vibration" or "whole-body vibration" or WBV or plyometric* or "aquatic training").mp.
8. 6 OR 7
9. 5 AND 8

Abbreviations and operational notes. AOP: arterial occlusion pressure; BFR: blood flow restriction; BFRT: blood-flow restriction training; MeSH: Medical Subject Headings; mp: multi-purpose field search in Ovid; TS: Topic Search. The strings above preserve the operational search record supplied for manuscript and PRISMA documentation.
