# Supplementary material for: Exercise Loading Strategies for Patellar Tendinopathy: A Systematic Review and Meta-Analysis of Clinical Outcomes
Source: J Funct Morphol Kinesiol. 2026 Aug 31;11(3):348. doi: 10.3390/jfmk11030348 (PMC13608764; doi:10.3390/jfmk11030348)
Supplement: Supplementary file 1 [file jfmk-11-00348-s001.zip › PRISMA 2020 Checklist.pdf]

PRISMA 2020 Checklist

Exercise Loading Strategies for Patellar Tendinopathy: A Systematic Review and Meta-Analysis of Clinical Outcomes

Checklist purpose: This completed checklist documents where each PRISMA 2020 item is reported in the manuscript and supplementary materials submitted for journal review.

Registration: PROSPERO CRD420261364763.

Section/topic	Item	PRISMA 2020 checklist item	Reported information	Status	Location in manuscript/supplement
TITLE	1	Title	Identify the report as a systematic review.	Yes	Title page: 'Exercise Loading Strategies for Patellar Tendinopathy: A Systematic Review and Meta-Analysis of Clinical Outcomes'.
ABSTRACT	2	Abstract	See the PRISMA 2020 for Abstracts checklist.	Yes	Abstract: Background, Objective, Design, Literature Search, Study Selection Criteria, Data Extraction and Synthesis, Results, Conclusion.
INTRODUCTION	3	Rationale	Describe the rationale for the review in the context of existing knowledge.	Yes	Introduction: paragraphs 1-4; explains clinical burden, existing evidence, gap in loading prescription/dose reporting, and need for focused synthesis.
INTRODUCTION	4	Objectives	Provide an explicit statement of the objective(s) or question(s) the review addresses.	Yes	End of Introduction: objective statement; primary outcome VISA-P and secondary outcomes specified.
METHODS	5	Eligibility criteria	Specify the inclusion and exclusion criteria for the review and how studies were grouped for the syntheses.	Yes	Methods: Eligibility Criteria; Population, Intervention, Comparator, Outcomes, Study design and publication characteristics; also explains primary quantitative versus qualitative/narrative evidence streams.
METHODS	6	Information sources	Specify all databases, registers, websites, organisations, reference lists and other sources searched or consulted to identify studies. Specify the date when each source was last searched or consulted.	Yes	Methods: Information Sources and Search Strategy; Supplementary Table S1. MEDLINE/PubMed, Web of Science Core Collection, Scopus, SPORTDiscus, and CENTRAL via Ovid/EBMR Reviews searched; database dates reported in Supplementary Table S1; reference lists screened manually.
METHODS	7	Search strategy	Present the full search strategies for all databases, registers and websites, including filters and limits used.	Yes	Supplementary Table S1: full electronic search strategies, platform/interface, filters/limits, dates, and records retrieved for MEDLINE/PubMed, Scopus, Web of Science Core Collection, SPORTDiscus, and CENTRAL/Ovid.
METHODS	8	Selection process	Specify the methods used to decide whether a study met the inclusion criteria, including how many reviewers screened each record/report, whether they worked independently, and how disagreements were resolved.	Yes	Methods: Study Selection; two reviewers independently screened titles/abstracts and full texts; disagreements resolved by discussion and third reviewer when required.
METHODS	9	Data collection process	Specify the methods used to collect data from reports, including how many reviewers collected data, whether they worked independently, processes for obtaining or confirming data from investigators, and automation tools if used.	Yes	Methods: Data Collection Process; two independent reviewers, standardized extraction form, author contact for unclear/unavailable data, third reviewer arbitration.

Section/topic	Item	PRISMA 2020 checklist item	Reported information	Status	Location in manuscript/supplement
METHODS	10a	Data items - outcomes	List and define all outcomes for which data were sought. Specify whether all results compatible with each outcome domain were sought and, if not, the methods used to decide which results to collect.	Yes	Methods: Eligibility Criteria / Outcomes and Outcomes sections; primary VISA-P/VISA final score; secondary pain, return to sport, satisfaction, adverse events, adherence, KOOS, Kujala, MVIC, CMJ, and 5-RM.
METHODS	10b	Data items - other variables	List and define all other variables for which data were sought. Describe assumptions made about missing or unclear information.	Yes	Methods: Data Collection Process; exercise dose variables including intensity, volume, frequency, contraction type, duration, progression, tempo, rest, supervision, adherence and co-interventions; NR/no author response rule; no imputation.
METHODS	11	Study risk of bias assessment	Specify the methods used to assess risk of bias in included studies, including tools, how many reviewers assessed each study, whether they worked independently, and how disagreements were resolved.	Yes	Methods: Risk of Bias Assessment; RoB 2 for randomized trials; role-specific appraisal/ROBINS-I-oriented classification for non-primary studies; Supplementary Table S3.
METHODS	12	Effect measures	Specify for each outcome the effect measure(s) used in the synthesis or presentation of results.	Yes	Methods: Data Synthesis; VISA-P/VISA final scores analysed as mean differences on the 0-100 scale; SMD and RR/OR planned if applicable; direction of effect defined.
METHODS	13a	Synthesis methods - eligibility for synthesis	Describe the processes used to decide which studies were eligible for each synthesis.	Yes	Methods: Data Synthesis; Table 1 final role in review; primary meta-analysis restricted to comparable active exercise/loading comparisons with extractable final-score data.
METHODS	13b	Synthesis methods - preparing data	Describe methods required to prepare the data for presentation or synthesis, such as handling missing summary statistics or data conversions.	Yes	Methods: Data Synthesis; SEM converted to SD using SD = SEM x square root of n; multi-arm trial handling; no imputation of unavailable data.
METHODS	13c	Synthesis methods - tabulation/visualisation	Describe methods used to tabulate or visually display results of individual studies and syntheses.	Yes	Results: Tables 1-6; Figure 1 PRISMA flow; Figure 2 forest plot; Supplementary Figures S1-S2; Supplementary Tables S1-S4.
METHODS	13d	Synthesis methods - statistical synthesis	Describe methods used to synthesize results and provide a rationale for model choice. Describe methods used to identify and explore heterogeneity.	Yes	Methods: Data Synthesis; random-effects REML with Hartung-Knapp adjustment; heterogeneity assessed using I2, tau2, and Cochran's Q; R/metafor specified.
METHODS	13e	Synthesis methods - sensitivity analyses	Describe methods used to explore possible causes of heterogeneity among study results, including subgroup analysis, meta-regression or sensitivity analyses.	Yes	Methods: Data Synthesis; subgroup analyses and meta-regression were prespecified but not conducted for the primary outcome because only six comparisons were available, making such analyses statistically unstable and clinically overinterpretable; non-primary studies synthesized narratively.
METHODS	13f	Synthesis methods - robustness	Describe any sensitivity analyses conducted to assess robustness of synthesized results.	Yes	Methods: Data Synthesis and Results: Narrative and Secondary Synthesis; explains why formal sensitivity analyses were not performed and how non-primary evidence was handled conservatively.
METHODS	14	Reporting bias assessment	Describe any methods used to assess risk of bias due to missing results in a synthesis.	Yes	Methods: Reporting Bias and Certainty of Evidence; funnel plot/asymmetry methods planned for >=10 studies; not

Section/topic	Item	PRISMA 2020 checklist item	Reported information	Status	Location in manuscript/supplement
					performed for primary synthesis because fewer than 10 comparisons contributed, so small-study effects remain an interpretive limitation.
METHODS	15	Certainty assessment	Describe methods used to assess certainty or confidence in the body of evidence for an outcome.	Yes	Methods: Reporting Bias and Certainty of Evidence; GRADE domains; Supplementary Table S4; Table 5 Summary of Findings.
RESULTS	16a	Study selection - search results	The study selection process is fully reported, including the separate CENTRAL/Ovid pre-screening handling and the transition from records to qualitative and quantitative synthesis.	Yes	Results: Study Selection; Figure 1 PRISMA flow diagram. 942 records identified; 463 duplicates/previously represented records removed; 115 CENTRAL/Ovid records outside the predefined completed journal-article screening subset documented as removed before screening; 364 records screened; 263 records excluded at title/abstract; 101 reports sought; 5 not retrieved; 96 assessed; 56 excluded; 40 retained.
RESULTS	16b	Study selection - excluded studies	Cite studies that might appear to meet the inclusion criteria but were excluded, and explain why they were excluded.	Yes	Results: Study Selection; Supplementary Table S2 provides article-level full-text exclusions with PRISMA-compatible reasons.
RESULTS	17	Study characteristics	Cite each included study and present its characteristics.	Yes	Results: Characteristics of Reports Included in the Qualitative Synthesis; Table 1; Table 2; all retained reports cited in text/tables.
RESULTS	18	Risk of bias in studies	Present assessments of risk of bias for each included study.	Yes	Results: Risk of Bias; Table 3 for six primary studies; Supplementary Figures S1-S2; Supplementary Table S3 for studies outside the primary meta-analysis.
RESULTS	19	Results of individual studies	For all outcomes, present summary statistics for each group and effect estimates with confidence intervals for each study, where applicable.	Yes	Results: Quantitative Synthesis; Table 4 presents study-level MDs and 95% CIs for VISA-P/VISA final score; Table 2 summarizes dose/intervention data.
RESULTS	20a	Results of syntheses - included studies	For each synthesis, briefly summarise the characteristics and risk of bias among contributing studies.	Yes	Results: Quantitative Synthesis; six active exercise/loading comparisons; Table 3 RoB 2 and Table 4 dataset/effects.
RESULTS	20b	Results of syntheses - statistical results	Present results of all statistical syntheses conducted. If meta-analysis was done, present summary estimate and precision, heterogeneity and direction of effect.	Yes	Results: Quantitative Synthesis; pooled MD 5.16 points, 95% CI 2.39 to 7.93; tau2=0.000; I2=0.0%; Q=3.50, p=0.624; Figure 2.
RESULTS	20c	Results of syntheses - heterogeneity	The manuscript reports statistical heterogeneity and explicitly distinguishes negligible statistical heterogeneity from clinically diverse active loading contrasts.	Yes	Results: Quantitative Synthesis and Discussion; explains that I2 should not be interpreted as evidence of clinical interchangeability among loading strategies.
RESULTS	20d	Results of syntheses - sensitivity analyses	Formal sensitivity analyses were not conducted for the primary outcome; the rationale is explicitly reported.	Not applicable / explained	Methods: Data Synthesis; Results: Narrative and Secondary Synthesis; Limitations. Non-primary studies had incompatible outcome structures, reporting formats, units of analysis, comparator types, or unresolved numerical extraction.
RESULTS	21	Reporting biases	Present assessments of risk of bias due to missing results for each synthesis assessed.	Yes	Results: Certainty of evidence for the primary outcome; Table 5; Supplementary Table S4; publication bias was not

Section/topic	Item	PRISMA 2020 checklist item	Reported information	Status	Location in manuscript/supplement
					formally assessed because fewer than 10 comparisons were available; small-study effects remain an interpretive limitation.
RESULTS	22	Certainty of evidence	Present assessments of certainty or confidence in the body of evidence for each outcome assessed.	Yes	Results: Certainty of evidence for primary outcome; Table 5; Supplementary Table S4; GRADE certainty low for VISA-P/VISA final score.
DISCUSSION	23a	Discussion - general interpretation	Provide a general interpretation of the results in the context of other evidence.	Yes	Discussion: paragraphs contrasting findings with Lim & Wong, Challoumas et al., Pavlova et al., and primary loading trials.
DISCUSSION	23b	Discussion - limitations of evidence	Discuss any limitations of the evidence included in the review.	Yes	Discussion: Risk of bias/certainty and Limitations paragraphs; low certainty, risk of bias, indirectness, small number of comparable comparisons, incomplete dose reporting.
DISCUSSION	23c	Discussion - limitations of review processes	Discuss any limitations of the review processes used.	Yes	Discussion: Limitations paragraph; Embase not searched because reproducible access/export could not be implemented; CENTRAL/Ovid screening used the completed journal-article subset; no robust publication-bias assessment due to k < 10; non-pooled secondary outcomes and unresolved numerical extraction reported.
DISCUSSION	23d	Discussion - implications	The manuscript discusses limitations in evidence completeness, heterogeneity, small-study effects, risk of bias, indirectness, and unavailable or unverified numerical data.	Yes	Discussion and Limitations: specifies the small number of comparisons, clinical heterogeneity despite negligible statistical heterogeneity, low certainty, inability to assess small-study effects formally, and quantitative exclusion of potentially relevant studies because of incompatible outcome structures or unavailable/unverified numerical data.
OTHER INFORMATION	24a	Registration and protocol - registration	Provide registration information for the review, including register name and registration number, or state that the review was not registered.	Yes	Methods: Study Design and Reporting Framework; PROSPERO CRD420261364763.
OTHER INFORMATION	24b	Registration and protocol - protocol access	Indicate where the review protocol can be accessed, or state that a protocol was not prepared.	Yes	Methods: Study Design and Reporting Framework; protocol prospectively registered in PROSPERO. Final amended record should be available through PROSPERO CRD420261364763.
OTHER INFORMATION	24c	Registration and protocol - amendments	Describe and explain any amendments to information provided at registration or in the protocol.	Yes	Methods: Information Sources and Search Strategy; Embase access/export limitation documented; CENTRAL/Ovid included as an electronic search source and did not add records requiring full-text retrieval or quantitative synthesis.
OTHER INFORMATION	25	Support	Describe sources of financial or non-financial support for the review and the role of funders or sponsors.	Yes	Declarations: Funding - This research received no external funding.

Section/topic	Item	PRISMA 2020 checklist item	Reported information	Status	Location in manuscript/supplement
OTHER INFORMATION	26	Competing interests	Declare any competing interests of review authors.	Yes	Declarations: Competing interests - the authors declare no competing interests.
OTHER INFORMATION	27	Availability of data, code and other materials	Report which data, analytic code and other materials are publicly available and where they can be found.	Yes	Declarations: Availability of data and materials; data are included in the article and supplementary materials. Table 4 provides primary meta-analysis dataset; Supplementary Tables S1-S4 provide supporting data.
