# Supplementary material for: Exercise Loading Strategies for Patellar Tendinopathy: A Systematic Review and Meta-Analysis of Clinical Outcomes
Source: J Funct Morphol Kinesiol. 2026 Aug 31;11(3):348. doi: 10.3390/jfmk11030348 (PMC13608764; doi:10.3390/jfmk11030348)
Supplement: Supplementary file 1 [file jfmk-11-00348-s001.zip › jfmk-4375337-supplementary figures S1_2.pdf]

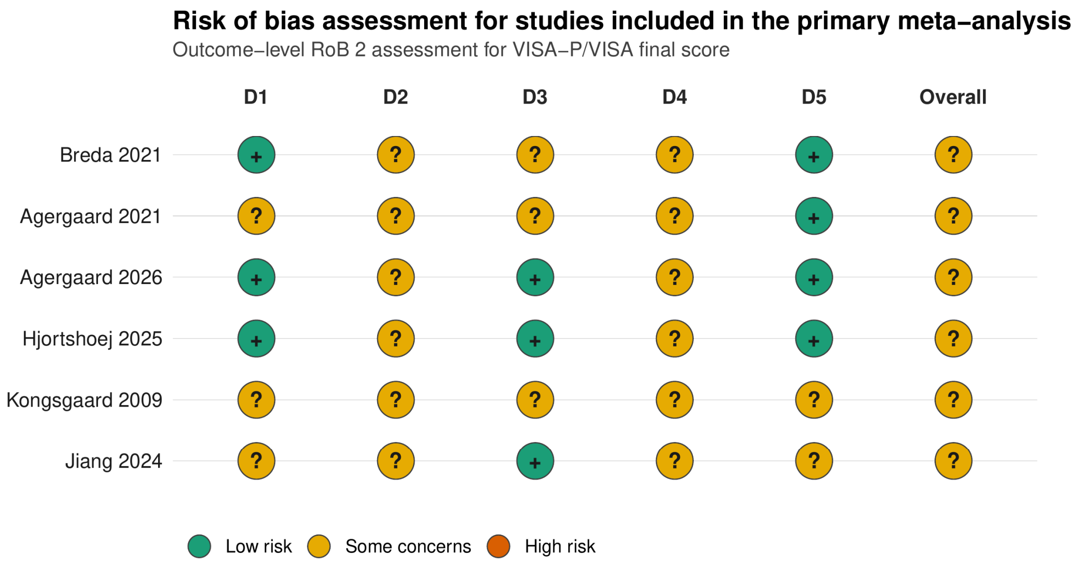

Supplementary Figure S1. Risk of bias assessment for studies included in the primary VISA-P/VISA meta-analysis. Risk of bias was assessed using the Cochrane Risk of Bias 2 tool at the outcome level, focusing on VISA-P/VISA final score at the primary post-intervention time point. D1: randomization process; D2: deviations from intended interventions; D3: missing outcome data; D4: measurement of the outcome; D5: selection of the reported result. Green indicates low risk of bias, yellow indicates some concerns, and orange indicates high risk of bias. All six studies were judged as having some concerns overall.

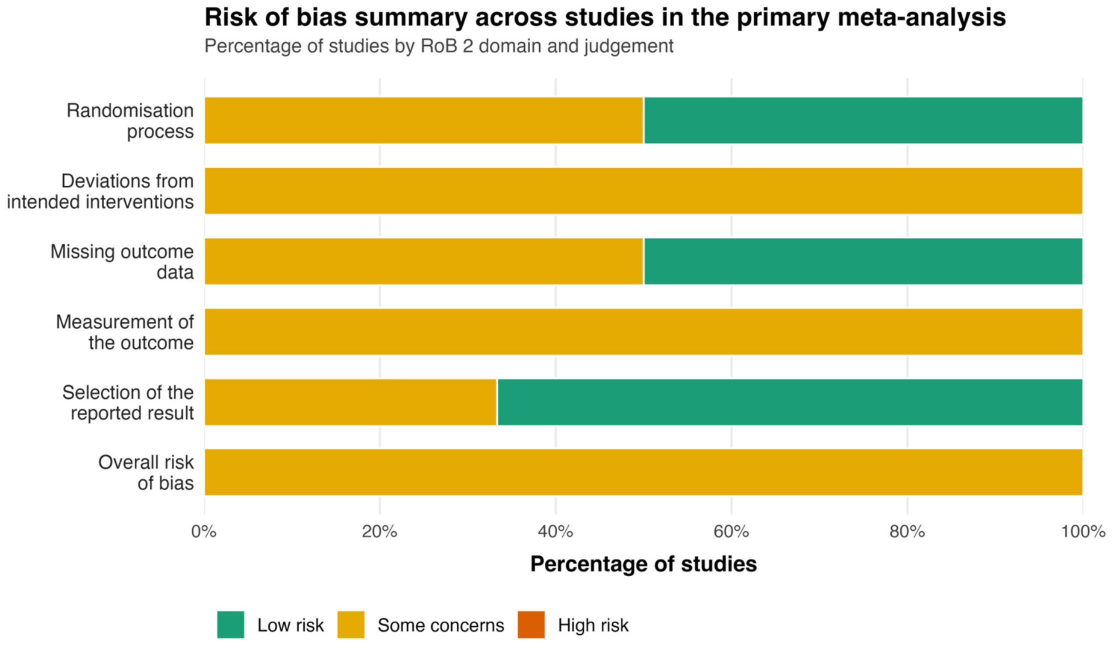

Supplementary Figure S2. Domain-level summary of RoB 2 judgements across studies included in the primary VISA-P/VISA meta-analysis. The plot shows the percentage of studies classified as low risk, some concerns, or high risk for each RoB 2 domain. No study was judged as high risk for the primary outcome.
